# Cerebral Organoids Derived from a Parkinson’s Patient Exhibit Unique Pathogenesis from Chikungunya Virus Infection When Compared to a Non-Parkinson’s Patient

**DOI:** 10.3390/pathogens10070913

**Published:** 2021-07-20

**Authors:** Emily M. Schultz, TyAnthony J. Jones, Sibei Xu, Dana D. Dean, Bernd Zechmann, Kelli L. Barr

**Affiliations:** 1 Department of Biology, Baylor University, Waco, TX 76706, USA; emily_schultz1@baylor.edu (E.M.S.); jtyanthony@gmail.com (T.J.J.); Julia_Xu1@baylor.edu (S.X.); dana_dean@baylor.edu (D.D.D.); 2 Center for Microscopy and Imaging, Baylor University, Waco, TX 76706, USA; Bernd_Zechmann@baylor.edu; 3 Center for Global Health and Infectious Disease Research, University of South Florida, Tampa, FL 33612, USA

**Keywords:** organoid, induced pluripotent stem cell, Parkinson’s disease, neurotransmission, neuroimmunity, neuroinflammation, Chikungunya, neuroinvasive

## Abstract

(1) Background: Arboviruses of medical and veterinary significance have been identified on all seven continents, with every human and animal population at risk for exposure. Like arboviruses, chronic neurodegenerative diseases, like Alzheimer’s and Parkinson’s disease, are found wherever there are humans. Significant differences in baseline gene and protein expression have been determined between human-induced pluripotent stem cell lines derived from non-Parkinson’s disease individuals and from individuals with Parkinson’s disease. It was hypothesized that these inherent differences could impact cerebral organoid responses to viral infection. (2) Methods: In this study, cerebral organoids from a non-Parkinson’s and Parkinson’s patient were infected with Chikungunya virus and observed for two weeks. (3) Results: Parkinson’s organoids lost mass and exhibited a differential antiviral response different from non-Parkinson’s organoids. Neurotransmission data from both infected non-Parkinson’s and Parkinson’s organoids had dysregulation of IL-1, IL-10, and IL-6. These cytokines are associated with mood and could be contributing to persistent depression seen in patients following CHIKV infection. Both organoid types had increased expression of CXCL10, which is linked to demyelination. (4) Conclusions: The differential antiviral response of Parkinson’s organoids compared with non-Parkinson’s organoids highlights the need for more research in neurotropic infections in a neurologically compromised host.

## 1. Introduction

There are over 100 medically relevant arboviruses recognized, and anyone who experiences an insect bite is at risk for exposure. Medically significant arboviruses are commonly found in the Togaviridae, Bunyaviridae, and Flaviviridae families. These families contain viruses that cause encephalitis or hemorrhagic fever, and vaccines or treatments are not available for most infections. Death rates are roughly 20,000–50,000 per year for any given arbovirus [1]. While mortality rates are proportionately low to number of cases, the risk of permanent disability is as high as 50% [1]. Rising temperatures and alterations in precipitation patterns have driven the emergence of these viruses into new regions [2,3,4,5]. Often, when these viruses emerge, new symptoms and increased pathology occur [6,7,8,9,10,11].

Infected individuals present with a spectrum of disease ranging from subclinical to death. The role of chronic diseases on intrinsic and innate immune defense is emerging as a significant player in a patient’s ability to respond to viral infections [12,13,14,15]. Studies have shown that those with a chronic condition like Parkinson’s disease (PD) can have increased oxidative stress, which can in turn compromise the immune system [16,17]. Other studies have shown that those with chronic neurological diseases specifically have altered inflammatory pathways, cytokine profiles, as well as autophagy dysregulation [18,19]. Clinical studies have shown that viral infections can induce expression of pro-inflammatory cytokines that can impact mood and neurocognitive performance [20,21,22]. Taken together, these studies show that the genetic background of the host can impact the severity and duration of sequelae.

Viral parkinsonism has been documented for a variety of human pathogens though there are few studies that evaluate the effects of viral infection on degenerative neurological diseases [1]. Post-viral parkinsonism has been documented for several viruses, including Dengue virus [23], West Nile virus [24], Japanese encephalitis virus [25], and St. Louis encephalitis virus [26].

How viruses cause parkinsonism is not known. Animal models of neurological infections do not translate to nor mimic changes in the human cerebral cortex documented in postmortem reports and imaging studies. Evaluation of central spinal fluid in human and rodent studies indicate that an inappropriate neuroimmune response is responsible [27]. This begs the question of how a virus might affect an individual with or without a predisposition to PD.

The use of human-induced pluripotent stem cells (hiPSC) in disease research is increasing not only because relevant data can be generated but because these cells can be differentiated systems that directly translate to a human model. Research on different hiPSCs and organoids has shown that products derived from patients with a disease state exhibit different morphology and gene expression compared with products derived from a normal patient [28,29,30,31]. This research has deepened the understanding of disease states and highlighted potential issues for diseased persons [31,32,33,34]. Studies have shown that neuronal hyperexcitability is found in organoids derived from patients with Alzheimer’s disease [35]. Unfortunately, most studies utilizing PD cerebral brain organoids evaluate morphology but not systemic differences in innate immunity and neurotransmission [36,37]. Most organoid modeling of PD is based on midbrain organoids that recapitulate PD pathologies of the dopaminergic networks, neurite disfunction, and abnormal localization of α-synuclein [37].

Since significant differences in gene and protein expression exist between hiPSC lines derived from individuals with and without PD, we hypothesized that these differences could impact response to viral infection [38]. This study aimed to classify those differences in response to infection with Chikungunya virus (CHIKV) so we might begin to understand how dysfunction in intrinsic and innate defenses could impact patient outcomes after infection with neurotropic arboviruses.

## 2. Results

### 2.1. Size and Morphology Do Not Differ between Organoids from Non-PD and Diseased Backgrounds in Response to CHIKV Infection

**Figure 1:**
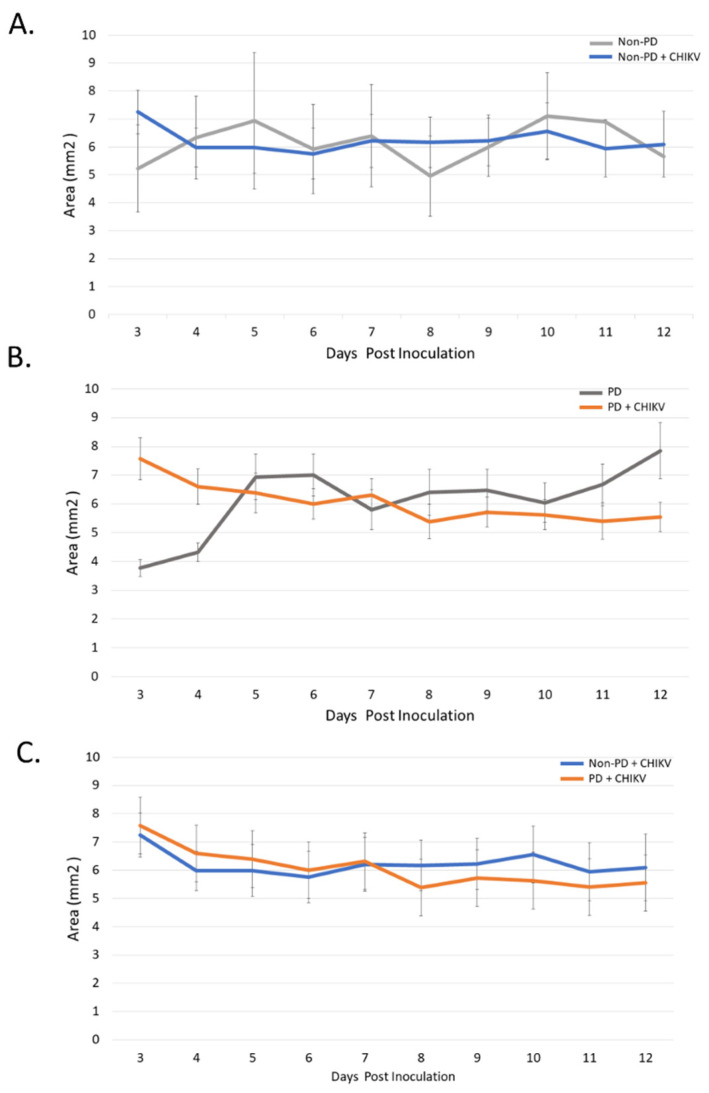
Impact of CHIKV on organoid size. Infected organoids were measured daily after inoculation. (**A**) Non-PD organoids had no significant change in size over 12 days when compared with the non-infected control. (**B**) PD organoids shrank an average of 0.6121 mm^2^ (*p* = 0.0149) at 12 days post inoculation compared with the non-infected control. (**C**) PD and non-PD organoids displayed similar reduction in size when infected with CHIKV.

The size of non-PD organoids did not significantly differ between the day of inoculation (6.2454 mm^2^) to the 12th day post infection (p.i.) (6.5338 mm^2^) (*p* = 0.2855) (Figure 1A). Over the course of 12 days, PD organoids lost 0.6121 mm^2^ (*p* = 0.0149), while all days of the experiment, PD and non-PD organoids did not differ in size (*p* = 0.0792–0.492) (Figure 1B,C). There were no significant differences in organoid size for either organoid types following infection with CHIKV (Figure 1C).

**Figure 2:**
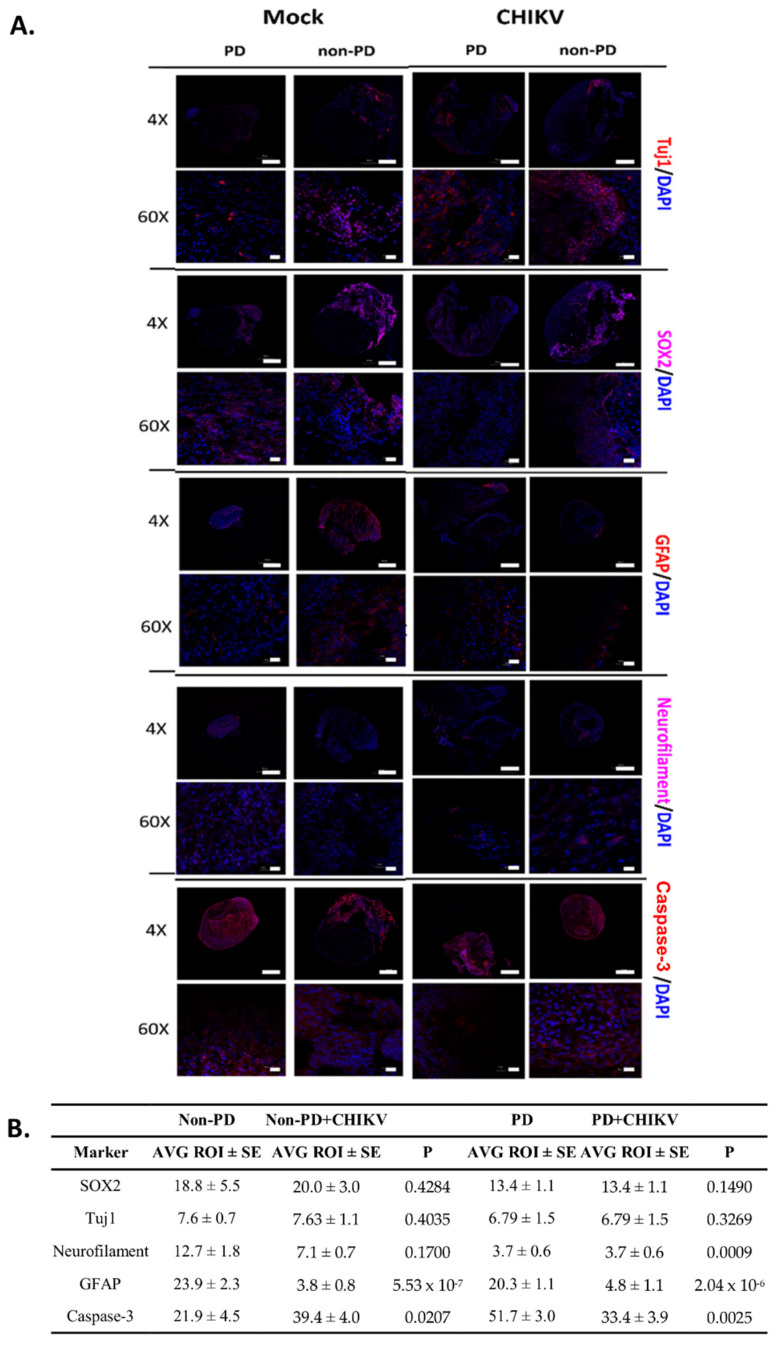
Immunofluorescence for morphology markers 14 days p.i. (**A**) Organoids were either infected with CHIKV in culture media or mock infected with plain culture media. DAPI was used throughout to visualize nuclei, and MAP2 was used to visualize microtubules to provide a structural reference. Images of organoids were obtained using an Olympus Fluoview 3000 confocal microscope. Scale bar represents 500 µm for images obtained at 4× magnification and 20 µm for images obtained at 60× magnification. CHIKV infection was confirmed via both RT-PCR and immunofluorescence with CHK-48 antibody on a separate slice from the same organoid. (**B**) Differences in fluorescence as determined by pairwise comparison of regions of interest. Organoids were fixed at 14 days p.i. Images of organoids were divided into 10 regions of interest (ROI). Average fluorescence for each ROI was determined, and then a Student’s *t*-test was used to compare PD with non-PD organoids. SE, standard error.

Immunofluorescence for morphology markers showed that both PD and non-PD organoids expressed sex-determining region Y-box 2 (SOX2), beta-III tubulin (Tuj1), neurofilament, and glial fibrillary acidic protein (GFAP). SOX2 is expressed in proliferating neural progenitors, and Tuj1 is a neuron-specific β-Tubulin. Both markers had increased fluorescence on the outer margins of the organoids, indicating growth of new neurons in both infected and control organoids (Figure 2A). GFAP is expressed by astrocytes and was found throughout mock-infected non-PD organoids, but PD organoids had less fluorescence (*p* = 0.0347) (Figure 2A,B). When CHIKV was present, significantly less GFAP was seen in both organoid types (*p* = 5.531 × 10^−7^ (non-PD), *p* = 2.046 × 10^−6^(PD)) (Figure 2A,B)). Caspase-3 was used to identify areas where development of brain tissue was present. Caspase-3 was seen in both PD and non-PD organoids with and without CHIKV (Figure 2A,B). Significantly more caspase-3 was detected in mock-infected PD organoids than in non-PD organoids (*p* = 0.0002) (Figure 2A,B). PD organoids exhibited decreased fluorescence, while non-PD organoids had increased fluorescence of caspase-3 (*p* = 0.00257 (PD), *p* = 0.02076 (non-PD)) (Figure 2A,B). When infected, both PD and non-PD organoids exhibited similar fluorescence patterns (*p* = 0.1213) (Figure 2A,B).

**Figure 3:**
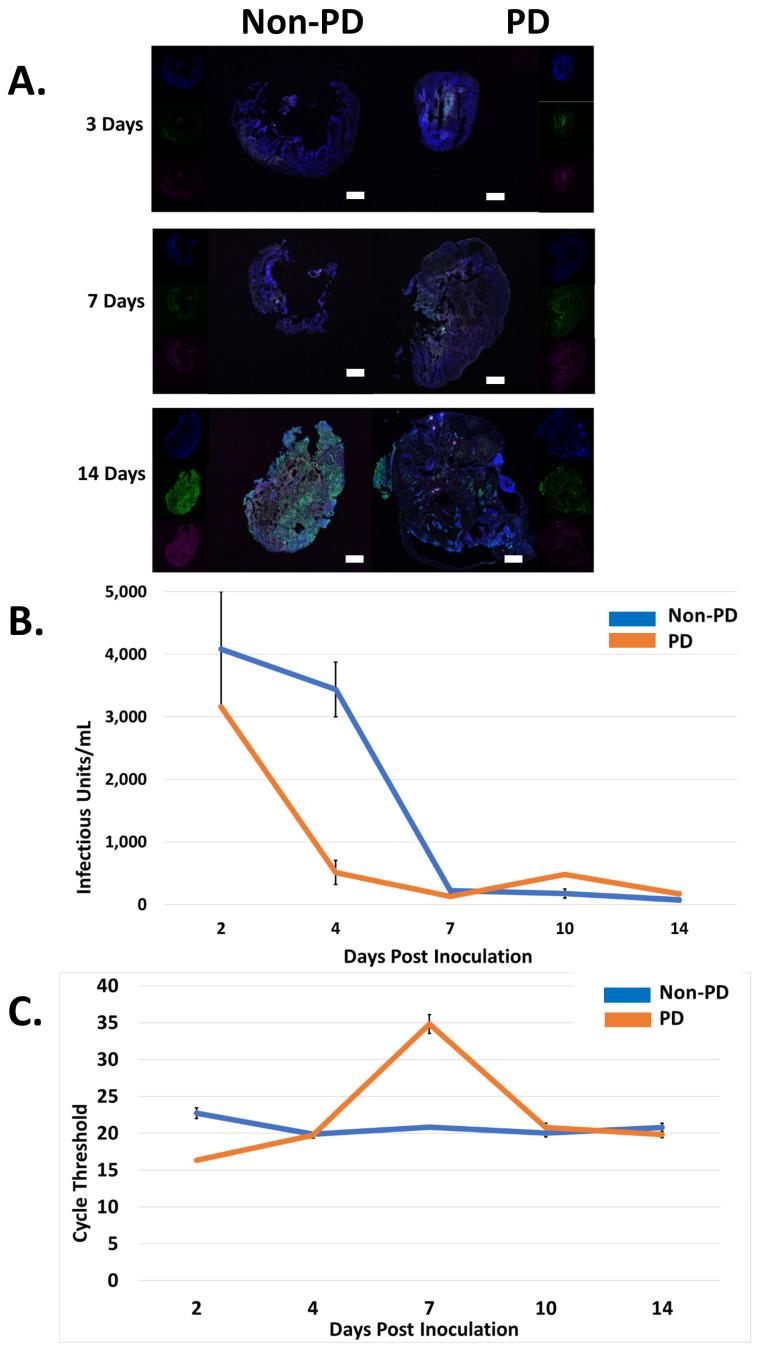
Chikungunya infection in PD and non-PD organoids. (**A**) DAPI was used throughout to visualize nuclei, microtubule-associated protein 2 (MAP2) was used to visualize microtubules to provide a structural reference, and CHK-48 was used to target the CHIKV E2 envelope protein. Images of organoids were obtained using an Olympus Fluoview 3000 confocal microscope. Scale bar represents 500 µm. (**B**) CHIKV titer, obtained from cell culture supernatant, decreased in both Non-PD and PD organoids, eventually becoming undetectable in PD and non-PD organoids after 7 days p.i., n = 3. (**C**) CHIKV, obtained from cell culture supernatant, was undetected via RT-PCR for both organoids types by 4 days p.i., n = 3.

Immunofluorescence was used to detect CHIKV over the course of 14 days. On days three and seven p.i., CHIKV was detected evenly distributed in both organoid types. However, at 14 days p.i., CHIKV remained evenly distributed in non-PD organoids but had developed foci of infection in PD organoids (Figure 3A). Significantly more CHIKV fluorescence was detected in non-PD organoids than PD organoids (*p* = 2.883 × 10^−7^) (Figure 3A). The viral titer, as determined by plaque assay of cell culture supernatant, drastically decreased within the first four days of CHIKV infection for both organoid types and by day five p.i., was undetectable (Figure 3B). RT-PCR of cell culture supernatant detected CHIKV nucleic acid from both organoid types for all 14 days p.i., with cycle thresholds ranging from 16–35 (Figure 3C).

### 2.2. Non-PD and PD Organoids Exhibit Unique Responses to CHIKV Infection

∆∆Ct comparison of Parkinson’s and Non-PD organoids with a non-infected non-PD control showed unique expression patterns for each organoid type. Of 208 genes with significant changes in expression, both organoid types had similar patterns of expression (i.e., up- or down-regulated) for 143 targets. A total of 65 genes displayed opposite patterns of differential expression for both organoid types.

### 2.3. Neurotransmission Is Reduced in Parkinson’s Organoids

**Figure 4:**
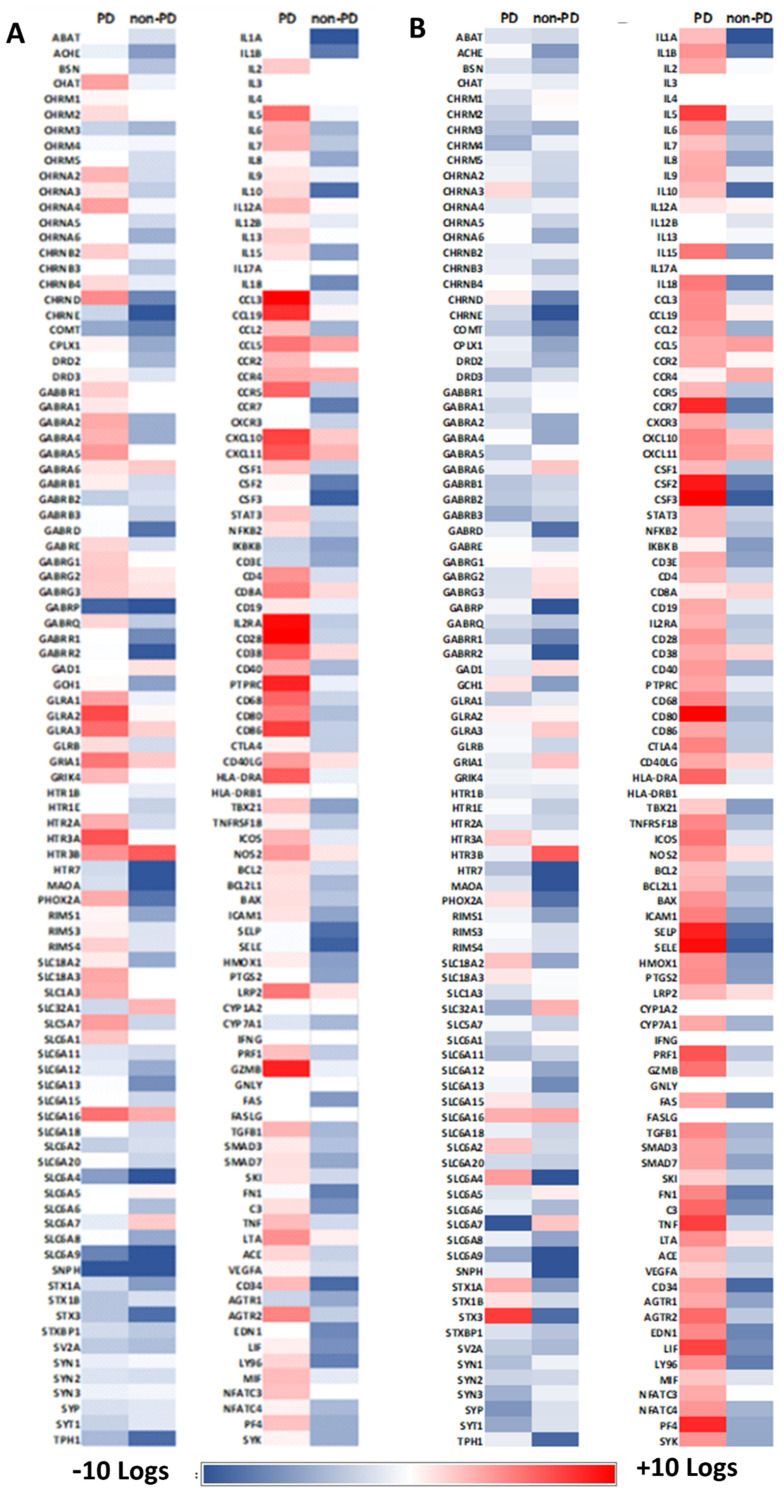
Differential expression of PD and non-PD organoids for genes associated with neurotransmission and immune response. RNA from 12 organoids per treatment was pooled and RT-PCR performed 14 days post inoculation. (**A**) ∆∆Ct analysis was performed where CHIKV-infected PD and non-PD organoids were compared to their respective non-infected control organoids. (**B**) ∆∆Ct analysis was performed where CHIKV-infected PD and non-PD organoids were compared to non-infected non-PD organoids.

∆∆Ct comparison of PD and non-PD organoids with their non-infected control showed that global expression of neurotransmitters was down-regulated in PD organoids in response to CHIKV (Figure 4A, Tables S1 and S2). The data show that non-PD organoids exhibited increased expression of all targets associated with the cholinergic, serotonin, dopaminergic, GABA, glycine, and glutamate neurotransmission (Figure 4A, Tables S1 and S2). Of note, PHOX2A, SLC6A4, STX1, and STX3 were up-regulated in PD organoids but down-regulated in non-PD organoids. SLC6A7 had the greatest amount of down-regulation, and STX3 had the greatest amount of up-regulation in PD organoids (Figure 4A, Tables S1 and S2). Non-PD organoids had the greatest up-regulation of HTR3B and the greatest down-regulation for GABARQ (Figure 4A, Table S1).

Since endogenous differences in expression exist for PD and non-PD cells [16,38,39], we performed ∆∆Ct analysis pairing the infected PD organoids with the non-PD control. Thus, we were able to compare how PD and non-PD organoids differ from non-PD organoids in the presence of CHIKV. Here, expression of dopamine receptors DRD2 and DRD3 was down-regulated in non-PD organoids −4.5 and −1.9 logs, respectively, but not differentially expressed in PD organoids in relation to the non-infected control (Figure 4B, Tables S1 and S3). Here, PDs organoids exhibited 3.5 log increased expression of dopamine receptor PHOX2A, while non-PD organoids had decreased expression by -8.27 logs, as determined by ∆∆Ct comparison (Figure 4B, Tables S1 and S3).

Overall, markers associated with cholinergic neurotransmission displayed decreased expression in non-PD organoids, while PD organoids tended to have no expression or increased expression of the same targets (Figure 4B, Tables S1 and S3). Of note, CHRNA2 and CHRND were up-regulated in PD organoids (3.15 and 4.72 logs, respectively), while non-PD organoids were down-regulated −2.37 and −7.25 logs (Figure 4B, Tables S1 and S3).

Fourteen GABA receptors displayed significant differential expression for non-PD organoids, and 13 receptors were differentially expressed in PD organoids (Figure 4B, Tables S1 and S3). Twelve GABA receptors were down-regulated in non-PD organoids both in relation to PD organoids and their non-infected control. GABRA2 was down-regulated −4.95 logs in non-PD organoids but up-regulated 3.56 logs in PD organoids (Figure 4B, Tables S1 and S3). While GABRP was down-regulated in both organoid types, PD organoids were down-regulated −9.09 logs, while non-PD organoids were down-regulated −13.2 (Figure 4B, Tables S1 and S3).

Four glycine receptors (GLRA1, GLRA2, GLRA3, and GLRB) showed increased expression for both PD organoids when compared with the non-PD non-infected control. Reduced expression was observed in non-PD organoids both in relation to housekeeping gene and in relation to PD organoids (Figure 4B, Tables S1 and S3). Both organoid types had decreased expression of GLS with PD organoids down-regulated −1.8 logs and non-PD organoids down-regulated −7.2 logs (Figure 4B, Tables S1 and S3).

Six glutamate receptors were evaluated, and PD organoids exhibited decreased expression of all targets over non-PD organoids when comparing their ∆∆Ct values to their respective non-infected controls (Figure 4A, Tables S1–S3). Of note, GRIN1 and GRIN2B was up-regulated in PD organoids (4.32 and 5.42 logs) but down-regulated in non-PD organoids −4.28 and −4.02 logs, respectively (Figure 4A, Tables S1 and S3).

Eight genes associated with serotonin neurotransmission were examined. Here, PD organoids displayed increased expression of HTR2A, HTR3A, and HTR3B and decreased expression of HTR7, MAOA, and TPH1 (Figure 4A, Table S3). In non-PD organoids, HTR2A was down-regulated −2.42 logs, HTR3A was not differentially expressed, and HTR3B was up-regulated 6.52 logs (Figure 4A, Table S1). HTR1B and HTR1E were down-regulated in non-PD organoids −1.46 and −2.98 logs, respectively, while these targets were not differentially expressed in PD organoids (Figure 4A, Tables S1 and S3).

Thirty-six targets representing a spectrum of transporters involved in neurotransmission were examined. ∆∆Ct values showed that non-PD organoids exhibited decreased expression of all targets except SLC6A16, SLC32A1, and SLC6A7 (Figure 4A, Table S1). ∆∆Ct values for PD organoids showed up-regulation of 5 SLC receptors that were down-regulated in non-PD organoids (Figure 4A, Tables S1 and S3). In non-PD organoids, RIMS 1, 3, and 4 were down-regulated, while only RIMS4 was differentially expressed in PD organoids at an increase of 2.13 logs (Figure 4A, Tables S1 and S3). Synaptophilin (SNPH); syntaxins (STX) 1A, 1B, and 3; and synapsins (SYN) 1, 2, and 3 were significantly down-regulated in PD organoids, while non-PD organoids were not- differentially expressed for SYN1 and SYN3 (Figure 4A, Tables S1 and S3). Synaptophysin (SYP) and synaptotagmin (SYT1) were also down-regulated in PD organoids more than 1 log when compared to infected non-PD organoids (Figure 4A, Tables S1 and S3).

**Figure 5:**
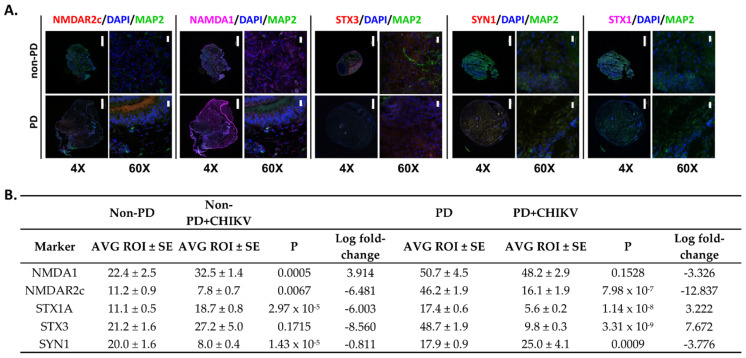
Immunofluorescence of neurotransmission markers of PD and non-PD organoids infected with CHIKV 14 days p.i. (**A**) Images were obtained to validate gene expression data. DAPI was used throughout to visualize nuclei, and MAP2 was used to visualize microtubules to provide a structural reference. Images of organoids were obtained using an Olympus Fluoview 3000 confocal microscope. Scale bar represents 500 µm for images obtained at 4× magnification and 20 µm for images obtained at 60× magnification. (**B**) Differences in fluorescence as determined by pairwise comparison of regions of interest. Images of organoids were divided in to 10 regions of interest (ROI). Average fluorescence for each ROI was determined, and then a Student’s *t*-test was used to compare PD with non-PD organoids. SE, standard error. Log fold-change values were obtained from Tables S1 and S2.

Immunofluorescence for neurotransmission markers for glutamate receptors NMDA1 and NMDAR2c indicated that PD organoids had stronger fluorescence of both markers compared to non-PD organoids (*p* = 0.00081, *p* = 0.000011) (Figure 5A,B). STX1 and STX3 fluorescence had significantly decreased expression in PD organoids (*p* = 1.148 × 10^−8^, *p* = 3.310 × 10^−9^) (Figure 5A,B). Non-PD organoids had significantly more expression of STX 1 (*p* = 2.976 × 10^−5^) but similar fluorescence with the mock-infected control (*p* = 0.17156) (Figure 5A,B). PD organoids also had increased fluorescence of SYN1 when compared to non-PD organoids, which had decreased expression (*p* = 0.00097, *p* = 1.431 × 10^−5^) (Figure 5A,B).

### 2.4. Alterations in Immune Regulation

Both PD and non-PD organoids were evaluated for immune response to CHIKV infection. Markers included surface receptors, stress response, oxidoreductases, cytokines including multiple chemokine receptors, and markers for cell lysis. Overall, PD organoids exhibited increased expression of all markers evaluated on the array when compared with their non-infected control (Figure 4A). ∆∆Ct analysis of both infected organoids types with the non-PD non-infected control showed a variable response for both organoid types. A total of 24 of 27 markers for surface receptors were up-regulated in PD organoids, including CD28 (10.11 logs), IL2RA (9.68 logs), and PTPRC (8.71 logs) (Figure 4B, Table S3). PD organoids showed decreased expression for CD4 (−2.26 logs), CD40 (−4.36 logs), CD28 (−2.88 logs), IL2RA (−3.3 logs), and PTPRC (−1.29) (Figure 4B, Table S1). Of note, CD34 was down-regulated in non-PD organoids -8.82 logs, but PD organoids were up-regulated 2.88 logs (Figure 4B, Tables S1 and S3). LY96 was down-regulated −7.55 logs in non-PD organoids but up-regulated 1.92 logs in PD organoids (Figure 4B, Tables S1 and S3).

Markers for stress response exhibited a similar pattern of expression for both organoids types though non-PD organoids were down-regulated −3.2 logs for AGTR2, while PD organoids were up-regulated 4.99 logs (Figure 4B, Tables S1 and S3). Further, C3 was up-regulated 1.51 logs in PD organoids but down-regulated in non-PD organoids −6.54 logs (Figure 4B, Tables S1 and S3). Non-PD organoids also exhibited significant down-regulation of colony-stimulating factors 1–3 as well as selectin E and P at a magnitude of at least 3 logs of PD organoids (Figure 4B, Tables S1 and S3). Oxidoreductases had a similar pattern of expression for both organoids type except HMOX1, which was up-regulated 1.02 logs in PD organoids but down-regulated −5.81 logs in non-PD organoids (Figure 4B, Tables S1 and S3).

Eight chemokine receptors were evaluated, and here, too, both organoid types had a similar expression pattern of up-regulation (Figure 4B). Of note, CCR5 was up-regulated 6.16 logs in PD organoids but down-regulated −3.4 logs in non-PD organoids (Figure 4B, Tables S1 and S3). CCR7 was down-regulated −7.82 logs in non-PD organoids but not differentially expressed in PD organoids (Figure 4B, Tables S1 and S3). PD organoids did not significantly express CXCR3 though non-PD organoids were down-regulated −3.03 logs (Figure 4B, Tables S1 and S3). Conversely, PD organoids were up-regulated 2.65 logs for PF4, but non-PD organoids were down-regulated −5.00 logs (Figure 4B, Tables S1 and S3).

Twenty-three surface receptors comprising a variety of interleukins, tumor necrosis factors, and chemokine ligands were evaluated. CCL2, CCL3, IL-6, IL-10, and TBX21 were significantly up-regulated in expression in PD organoids but down-regulated in non-PD organoids (Figure 4B, Tables S1 and S3). IL-1A, IL-1B, and IKBKB were down-regulated for both organoid types (Figure 4B, Tables S1 and S3). Both GZMB and PRF1 up-regulated in PD organoids but down-regulated in non-PD organoids (Figure 4B, Tables S1 and S3).

**Figure 6:**
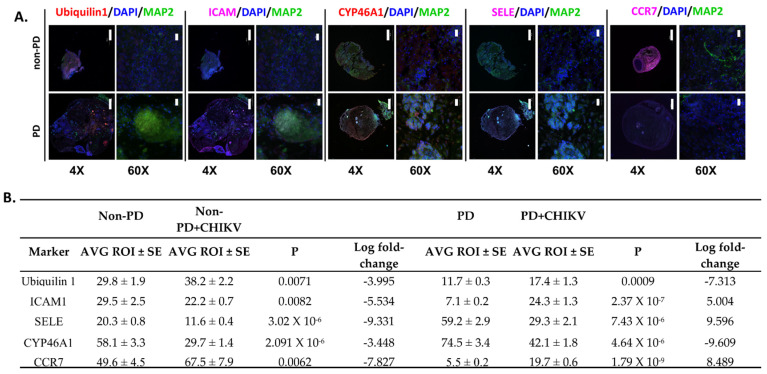
Immunofluorescence of immune markers in PD and non-PD organoids infected with CHIKV 14 days p.i. (**A**) DAPI was used throughout to visualize nuclei, and MAP2 was used to visualize microtubules to provide a structural reference. Images of organoids were obtained using an Olympus Fluoview 3000 confocal microscope. Scale bar represents 500 µm for images obtained at 4× magnification and 20 µm for images obtained at 60× magnification. (**B**) Differences in fluorescence as determined by pairwise comparison of regions of interest. Images of organoids were divided in to 10 regions of interest (ROI). Average fluorescence for each ROI was determined and then a Student’s *t*-test was used to compare PD with non-PD organoids. SE, standard error. Log fold-change values were obtained from Tables S1 and S2.

Immunofluorescence for immunological markers showed that ubiquilin and CCR7 had significantly less fluorescence in infected PD organoids than infected non-PD organoids (*p* = 2.115 × 10^−6^, *p* = 9.004 × 10^−5^) (Figure 6A,B). SELE had decreased fluorescence in infected PD and non-PD organoids, which reflects gene expression data (*p* = 7.435 × 10^−6^, *p* = 3.028 × 10^−6^) (Figure 6A,B). CYP46 fluorescence was also reduced in CHIKV-infected organoids (*p* = 2.091 × 10^−6^, *p* = 4.645 × 10^−6^) (Figure 6A,B, Tables S1 and S3). ICAM fluorescence was significantly reduced in CHIKV-infected non-PD organoids (*p* = 0.00822) but significantly increased in PD organoids (*p* = 2.376 × 10^−7^) (Figure 6, Table 1, Tables S1 and S3).

### 2.5. Expression for other Markers

Additional markers were evaluated to determine validated gene expression and immunofluorescence data and to measure the expression of markers associated with PD, especially in the mid-brain and mid-brain organoids [40,41]. There was no significant difference in GFAP expression (*p* = 0.3789), while PD organoids had increased expression of Iba1 (*p* = 0.0004) (Table 1). CRYM, TH, and GBA were evaluated, as these genes have been shown to be differentially expressed in Parkinson’s models [42,43,44]. Here, PD organoids had less expression of TH (*p* = 0.002), GBA (*p* = 0.0014), and CRYM (*p* = 0.0002) (Table 1). DRD1 was not included in the gene expression arrays; however, since it is the main dopamine receptor in the brain, we evaluated its expression. Here, average Ct was 27.6, while PD organoids average Ct was 28.5 (*p* = 0.0281) (Table 1). CCR5, IL-1a, IL-10, CXCL10, HTR3B, and SLC6A4 were chosen to validate gene expression array data. When CHIKV was present, CCR5 expression was up-regulated in non-PD organoids, which reflects the gene expression studies (*p* = 0.019) (Table 1, Table S3). PD organoids were down-regulated for CCR5 via RT-PCR but up-regulated in the gene expression studies (Table 1, Table S3). For the remaining targets, CXCL10 and HTR3B reflected gene expression data, while IL-1, IL-10, and SLC6A4 exhibited opposite expression compared with the gene expression data (Table 1, Table S3).

## 3. Discussion

Research of viral encephalitis and other viral infections of the CNS are crippled by necessary ethical restraints. This field relies on autopsy findings, which are then typically applied to rodent models. Rodents do not present with symptoms of CNS pathologies unless they are genetically modified to be immune-deficient or have large quantities of virus administered via intracerebral injection or injection into other parts of the CNS. This has provided useful insights into the pathogenesis of these viruses but unfortunately has not translated to treatment or prevention of human disease.

While animals are valid and useful models, organoids could serve as a preliminary platform for screening that can better inform the design of animal studies and choice of genetic background. Ongoing advances with stem-cell research have provided a platform for producing specific cell types or organoids from human stem cells. Within the last few years, significant advances in human health have been made using stem cells and organoids [45,46,47,48,49]. Human stem cells and organoids are emerging as a useful tool for virus research [50,51]. Not only do they replicate cellular composition and expression of humans, but they are also less expensive, safer, and easier to use than animals. Using organoids as a model can also produce better data due to the ability to have more replicates and numbers per treatment.

When infected with CHIKV, non-PD and PD organoids produced similar amounts of virus for the same period, but after two weeks post-infection, PD organoids started to shrink. Immunofluorescence showed unique virus distribution patterns for non-PD and PD organoids. While non-PD organoids displayed uniform distribution of the CHIKV E2 protein, PD organoids exhibited local accumulation of CHIKV E2. This matches postmortem and necropsy data showing focal distribution of West Nile virus in brain tissue [52,53]. Unfortunately, histological CNS data from CHIKV-infected humans is not available since the role of CHIKV in neurological disease is an emerging topic [6,54,55]. This leads to the question of if the altered inflammatory response associated with PD contributes to the distribution of virus in the CNS and contributes to the establishment of chronic infection. In mice, CHIKV evades the CD8^+^ T-cell response to establish persistent infections [56]. Gene expression data show that IL-12 and IL-18 activate the CD8^+^ T-cell response [56]. It could be that the reduced expression of IL-12 and IL-18 in CHIKV-infected non-PD organoids is contributing to the infection patterns we observed. CD4 and CD8 cells would need to be incorporated into this model to delineate how the innate immune response is impacting the activation of T cells.

The distribution and density of astrocytes in non-infected organoids is comparable to other studies that document organoid morphology [57,58]. GFAP was used to detect activated astrocytes. Histological studies have found that many mature astrocytes do not express significant GFAP unless activated [59]. Thus, the lack of staining observed in non-infected non-PD organoids is likely indicative that astrocytes are in an inactivated state. Staining of PD organoids showed extensive distribution of astrocytes with a section of astrogliosis. Non-PD infected organoids had astrocytes distributed solely on the outer margins, which mimics other studies’ investigation astrocyte activation in organoids [57]. Neurofilament is a component of mature neuronal cytoskeleton often found in high concentrations in axons. In growing or developing neurons, neurofilament may not be readily apparent since younger axons are much smaller than mature neurons [60]. Imaging studies show neurofilament staining for all organoid types, which suggests that organoids possess mature neuron populations. Statistical analysis was not performed for these images due to the heterogeneous distribution of cell types (neuronal, glial, ependymal, etc.) between organoids.

SOX2 and Tuj1 were used to observe neuron proliferation in response to CHIKV. When compared to non-infected organoids, SOX2 fluorescence was similar in CHIKV-infected non-PD organoids but was reduced in CHIKV-infected PD organoids. SOX2 is a transcription factor that regulates pluripotency and neurogenesis and is integral to the growth and repair of neurons [61]. The reduced expression of SOX2 in infected PD organoids could mean that there is dysfunction in neuron growth; however, detection of other assorted transcription factors would be necessary to understand the expression pattern.

∆∆Ct was used to analyze changes in gene expression. When infected organoids were compared to their respective non-infected controls, PD organoids showed a pattern of up-regulation, while non-PD organoids showed a pattern of down-regulation. While this is interesting in and of itself, this analysis did not provide much insight as to whether PD organoids were mounting an antiviral response that reflected the response of non-PD organoids. Previous work has documented that endogenous expression of most genes is different for PD and non-PD cells and patients [16,38]. Thus, any changes, or lack thereof of PD organoids to viral insult may not reflect a typical antiviral response when compared to its non-infected counterpart. When ∆∆Ct analysis was performed comparing infected PD organoids with a non-PD non-infected control, it was observed that both non-PD and PD organoids had similar expression patterns for most markers in response to CHIKV infection. This indicates that PD organoids modify their expression in response to virus infection for most markers and mount a response like non-PD organoids.

This study showed an overall pattern of excitation of GABA, glycine, glutamate, and serotonin receptors in PD organoids when infected with CHIKV, while non-PD organoids exhibited a decreased pattern of expression for the same markers. While it is well documented that CHIKV can cause long-term or permanent depressive sequelae, there are no studies describing changes in neurotransmission. Two studies have reported the potential antiviral activity of serotonergic drugs on CHIKV replication though viral inhibition assays though impacts on serotonin neurotransmission are not described [62,63]. GABRP, HTR3, SLC6A4, SLCA9, and SNPH were differentially expressed for both organoid types. HTR3 receptors are associated with neuro-gastrointestinal and psychiatric conditions that are controlled with 5-HT3 receptor antagonists [64].

While the gene expression data shows that the response to CHIKV involves changes in neurotransmission and immune response, clinical trials have shown that dysregulation of the inflammatory response can remodel neurotransmission, leading the changes in mood and cognition [13,65,66]. The reduced expression of ACHE has been linked to depression and cognitive deficits in human studies [67,68,69]. Perhaps ACHE could be contributing to persistent depressive sequalae in CHIKV patients.

The data show that both PD organoids have increased expression of NFKB2, a transcription control protein that functions in the innate antiviral response [70]. In PD, NFKB2 is activated with IL-17 when cultured with T-lymphocytes [71]. Activation of NFKB2 results in the production of interferons, which play a significant role in the innate antiviral response [70]. We also observed up-regulation of many proinflammatory cytokines in response to CHIKV infection in PD organoids when compared with non-PD organoids. CSF2 and CSF3 respond to infection by inducing inflammation and recruiting lymphocytes to the site of infection. Several viruses evade this immune response by blocking autophagy and thereby blocking monocyte differentiation and apoptosis [72,73].

Oxidoreductases determine MHC class I surface exposure and influence the activation of inflammation cascades. When found on the plasma membrane, oxidoreductases signal intracellular stress status to the immune system [74]. In particular, HMOX1 has antiviral activity with increased levels associated with clearance of infection [75,76,77]. Our data show that PD organoids had increased expression of HMOX1, while non-PD organoids had decreased expression. When present, HMOX1 interacts with IL-10 (also down-regulated in infected non-PD organoids) as an anti-inflammatory mechanism of the innate immune response [78]. Research has shown that Zika and Dengue viruses decrease host expression of HMOX1 as part of their antiviral response [75,79]. The deficit of HMOX1 could be contributing to the persistence and pattern of CHIKV in the organoids.

Cytokines direct the innate immune response and play an important role in regulating the adaptive immune response. Specific cytokines can serve as biomarkers for viral infections [80]. Our data support that PD organoids have increased expression of IL-5, IL-6, and IL-10, indicating that there could be an activation of a Th2 response. However, IL-12 was also significantly up-regulated in PD organoids, which would favor a cell-mediated inflammatory response to stress or infection as well as the activation of cytotoxic T lymphocytes. We observed significant down-regulation of IL-18 and IL-1B in non-PD organoids. These interleukins catalyze the production of several proinflammatory cytokines and recruit immune cells to the site of microbial infections. IL-12 promotes protective immunity to a variety of viruses, and IL-12 and IL-18 work together during the antiviral response [81]. With IL-12 up-regulated and expression of IL-18 and IL-1 down-regulated, both PD and non-PD organoids could be having dysfunctional antiviral response. Of note, non-PD organoids had down regulation of IL-1 and IL-10 in response to CHIKV. This aligns with studies that have shown that reduced expression of IL-1 and IL-10 can exacerbate mental illness or psychotic episodes following infection with CHIKV [82,83,84].

Chemokines are a subset of cytokines that are activated in response to tissue damage as well as foreign proteins and antigens. Overproduction of chemokines is associated with a variety of autoimmune diseases. Most chemokines we examined were expressed at greater levels in PD than non-PD organoids at 14 days post infection. This state of inflammation could potentially cause complications for responding to viral infections. CCL19 is a chemokine that binds to the CCR7 receptor and acts to recruit dendritic cells. CCL19 was up-regulated in PD and non-PD organoids. CCR7 was down-regulated in non-PD organoids but up-regulated in PD organoids. The expression profiles of PD organoids reflect expression profiles documented from cerebrospinal fluid from patients infected with Varicella–Zoster virus [85]. Also, studies in CCR7-deficient mice reported increased death from West Nile virus infection via over-recruitment of leukocytes and inflammation [86]. The reduced activity of CCR7 we observed could render organoids vulnerable to neuropathogens due to enhanced expression towards an inflammatory response.

CCL3 interacts with CCR4 and CCR5 during the acute inflammatory response and functions to recruit monocytes, which can have an impact on neuroimmunity [87]. The increased expression of CCL3 and CCR5 in both PD and non-PD organoids also occurs during infection with respiratory pathogens and is associated with severe manifestations of disease [88]. Animal studies support that expression of CCR5 is up-regulated in CNS infections with Japanese encephalitis virus and positively correlated with increased pathogenesis [89]. Work has shown that increased levels of CCR5 contribute to demyelination and CNS disease [90]. The increased expression of CCR5 in PD organoids indicates that they are in an inflammatory state or could be experiencing neuronal damage.

The complement system is a part of the innate immune response that can lyse cells, activate inflammation, target virus to phagocytic cells, and clear non-cytopathic viruses from the circulatory system. Here, we evaluated the expression of C3 as it functions in both classical and alternative complement activation pathways, and deficiency of C3 can make humans more susceptible to viral and bacterial infections [91,92]. In our study, PD organoids had increased in C3 expression compared with non-PD organoids, suggesting a functional complement system. Non-PD organoids had down-regulation of C3, which has been reported in patients with hepatitis C infection [93]. Functional expression of C3 is necessary to neutralize West Nile and other viruses which cause acute neurological infections and death [94,95]. This poses an important question to be addressed in future research: could a reduction in C3 leave patients with CHIKV disease primed for neurological sequalae? Another future direction to build upon this study would be to conduct gain/loss of function studies on specific genes. Currently, disease modeling and the methodologies for conferring gain/loss of function in organoids of disease and non-diseased nature while also infected with a pathogen has not progressed to the point of reliability and reproducibility. This study presents key genes with altered expression and provides a foundation for these methodologies and studies to further develop.

The use of only two cell lines (one non-PD, one PD) is a limitation of this study due to the extensive genetic variation of PD. There are nearly 400 hiPSC cell lines derived from PD patients available for research [23]. While typical neuronal studies utilize up to five cell lines per study (three diseased, two control), the appropriate numbers of cell lines to use for brain organoid research is still under debate [23]. PD research utilizing organoids typically differentiate from one non-PD and one PD hiPSC line [47]. In depth analysis of preliminary concepts requires substantial resources and time that is not justifiable for pilot studies, especially when generating organoids [23]. Thus, preliminary data is often limited to two cell lines (control and diseased) [94,95,96]. Regardless, the findings here need substantiation in organoids derived from additional cell lines. Of the two cell lines used in this study, one was obtained from the foreskin of a newborn, and one was obtained from a male donor aged 63 years. Studies in rodents have found age-related impact of viral infections, and observational studies in humans have reported specific pathogenesis in neonates [97,98]. These studies above are reflective of whole organisms where DNA damage and telomere shortening are present in all somatic cells. Whole organisms also have functional immune systems that can contribute to pathogenesis. A benefit of hiPSC is that donor age does not affect the expression markers in differentiated cells since cellular rejuvenation occurs during the reprogramming of somatic cells into stem cells [99,100,101].

## 4. Materials and Methods

### 4.1. Cell Culture and Virus Propagation

Two cell lines were cultured: human-induced pluripotent stem cells (ACS-1019: ATCC, Manassas, VA, USA) and human-induced pluripotent stem cells (hiPSC) with Parkinson’s disease (ACS-1013: ATCC, Manassas, VA, USA), which has mutations at the marker for tyrosine hydroxylase 1 [102]. The exposure of the donors to arboviruses is unknown. Both cell lines were both cultured in mTeSR1 media (StemCell Technologies Cat #85850) on plates coated with vitronectin XF (Stemcell Technologies Cat #07810 and #07183) prior to organoid formation. *Cercopithecus aethiops* kidney cell line Vero E6 (ATCC CRL-1586) were grown in Dulbecco’s modified Eagle’s medium (DMEM) with 10% FBS, supplemented with penicillin/streptomycin, 1× non-essential amino acids, 1× Glutamax, and 1 mM HEPES. All cell lines were incubated at 37 °C/5% CO_2_. CHIKV (181/25) was obtained from BEI Resources (NR-50345) and expanded once in Vero E6 cells in a biosafety level 2 laboratory. This specific strain of CHIKV, which is a live attenuated strain derived from a human isolate in Asia, was selected based on recent peer-reviewed reports for CHIKV persistence in tissues and use in stem-cell models [56,103,104,105]. Our goal was to achieve long-term infection without significant loss of organoid tissue. While CHIKV 181/25 is attenuated, it still produces disease in cell culture and humans, which prevented its use as a vaccine [104,105,106]. Infectious units (PFU) and viral titers were measured via plaque assay.

### 4.2. Generation and Infection of Human Cerebral Organoids

For this preliminary, observational study, cerebral organoids were utilized because they have cortical neurons that contain functional synapses that produce Ca^+^ surges with glutamate release, which can be affected when a virus is present [107]. Cerebral organoids were also chosen because they develop immunocompetent astrocytes that are key players in neuroinvasive disease response [58]. While mid-brain organoids are standard for PD research, they omit the cerebral cortex, which is integral to viral pathogenesis in the human CNS [108]. Cerebral organoids were formed from hiPSC ACS-1019 and hiPSC ACS-1013 using the StemDiff Cerebral Organoid Kit (StemCell Technologies Cat #08570) and StemDiff Cerebral Organoid Maturation Kit (StemCell Technologies Cat. #08571), following the manufacturer’s directions. This methodology has been used for exploring pathologies for Alzheimer’s disease [35], brain development [109], and a host of other applications [110]. Briefly, hiPSC were harvested with Gentle Cell Dissociation Reagent (StemCell Technologies Cat #07174) and then seeded into ultra-low attachment 96-well plates (Corning Cat #7007) at a density of 9000 cells/well. Cells were seeded in seeding media containing Y-27632. On days 2 and 4, 100 uL of Embryoid Body (EB) formation media was added to the wells. On day 5, EBs were observed to be rounded and tightly packed spheres about 200nm in size. EBs were embedded in Matrigel and incubated at 37 °C for 1 h. Embedded EBs were then placed in a 6-well, ultra-low attachment plate (StemCell Technologies Cat #3471) containing organoid expansion media. After 3 days, media was changed to maturation media. Media changes then occurred twice per week, and cerebral organoids were matured for 53 days before data collection to allow for full maturation and to best resemble an adult brain [32,111].

At 53 days, organoids were transferred to ultra-low attachment 24-well plates at 1 organoid per well. Organoids were infected with 100,000 (~MOI 0.001) PFU per well. Controls included mock-infected cerebral organoids. Supernatant was taken at 48 h, 4 days, 7 days, 10 days, and 14 days post infection and pooled amongst similar treatments. Samples of cerebral organoid tissue were also taken at 48 h, 3 days, 7 days, and 14 days post infection, and preserved in 4% paraformaldehyde solution in PBS (ThermoScientific Cat# J19943-K2) at 4 °C.

### 4.3. Viral Quantification

Plaque assays were performed using the pooled supernatant samples from each treatment at each time point taken during the experiment, following methods described elsewhere (Barr et al. [6]. Briefly, serial dilutions of virus in PBS were inoculated onto confluent Vero E6 cells and covered with 0.25% methylcellulose overlay. After 3 days, the overlay was removed, and cells were stained with Coomassie blue. For quantitative real-time PCR, RNA was extracted from all collected samples (3 replicates each) using a kit in accordance with the manufacturer’s instructions (Zymo Quick Viral RNA kit Cat #R1034). Virus was measured using Verso One-Step RT-qPCR Kit, SYBR Green, ROX (Thermo Fisher Cat#AB4100A) and primers specific for the CHIKV E1 gene [112].

### 4.4. Cerebral Organoid mRNA Extraction and Gene Expression

mRNA was extracted using Zymo Quick-RNA Kit (Zymo Research Cat #R1052), and cDNA was generated using Applied Biosystems High-Capacity cDNA Reverse Transcription Kit (Applied Biosystems #4368814). Gene expression studies were then conducted using TaqMan Array Human Neurotransmitters (Applied Biosystems Cat #4414094), TaqMan Array Human Immune Response (Applied Biosystems #4414204), and TaqMan Array Human Alzheimer’s Disease (Applied Biosystems Cat #4414070) with Applied Biosystems TaqMan Gene Expression Master Mix (Applied Biosystems Cat #4369016). Results were analyzed using the ∆∆CT method.

Additional RT-PCR was performed to validate gene expression data and to measure expression of astrocytes, microglia, and markers associated with Parkinson’s disease. RNA and cDNA were obtained as described above. RT-PCR was performed using PowerTrack SYBR Green Master Mix (ThermoFisher Cat #A46012), per manufacturer’s instructions. Primers were designed in Primerquest (IDT SciTools) from human transcripts obtained NCBI Nucleotide. RT-PCR primers were designed for glial fibrillary acidic protein (GFAP), ionized calcium binding adaptor molecule 1 (Iba1), chemokine receptor 5 (CCR5), dopamine receptor D1 (DRD1), tyrosine hydroxylase (TH), crystallin mu (Crym), and glucosylceramidase beta (GBA) (Table 2). DRD1 is the most common dopamine receptor in the CNS. GBA mutations are a common cause of PD, and expression of GBA is reduced in idiopathic and other genotypes of PD patients [44]. TH is found throughout the brain but in high concentrations in dopaminergic neurons located in the nigrostriatal region [43]. In PD, TH is phosphorylated and degraded, leading to TH deficiency [113]. Crym binds NADPH and is found at high concentrations in the brain, heart, and kidneys [114]. Reduction of Crym expression has been linked to striatal degeneration and the dysregulation of TH [115]. Aldh1l1 was evaluated as a second astrocyte marker due to the heterogeneity in CNS tissue [116].

### 4.5. Cerebral Organoid Size Measurements

Organoids were imaged using ImageQuant LAS 4000 with the bright field filter under high resolution with automatic exposure. Organoid size was determined by using ImageJ (National Institutes of Health). The scale of the program was set to 13.9327 pixels/mm, and the area of each organoid was recorded. Results are expressed as an average between at least 12 organoids per treatment. ANOVA was performed to determine significance between PD and non-PD organoids. A Student’s *t*-test was used to identify significance between organoids size pre-inoculation and at 13 days post infection.

### 4.6. Immunofluorescence

Immunofluorescence was used to validate gene expression and PCR data. Organoids were fixed in 4% paraformaldehyde in PBS (ThermoScientific Cat# J19943-K2) overnight at 4 °C and then cryoprotected in 30% sucrose prior to sectioning. After freezing samples at −80 °C, organoid sections of 18 micrometers thick were produced using a cryomicrotome (CryoStar NX50, Thermo Fisher Scientific, Waltham, MA, USA). Afterwards, organoid sections were blocked in 5% fetal sheep serum, and primary staining was conducted overnight at 4 °C (Table 3). Secondary staining was then conducted using fluorescent antibodies (Table 3) for 1 h at 25 °C. Slides were mounted with ProLong Gold Antifade Reagent with DAPI (Cell Signaling Technology #8961S). DAPI was used throughout to visualize nuclei, and MAP2 was used to visualize microtubules to provide a structural reference. Chikungunya E2 monoclonal antibody CHK-48 (NR-44002) was obtained from BEI resources and used to visualize CHIKV. CHIKV infection of other images was confirmed via both RT-PCR and immunofluorescence with CHK-48 antibody on a separate slice from the same organoid. Organoids were imaged using an Olympus Fluoview 3000 Confocal Laser Scanning Microscope (Olympus America Inc., Center Valley, PA, USA). All images were obtained using the same parameters including slices, laser power, gain, and offset. Statistically significant differences in image fluorescence were then determined using the computer program cellSens (Olympus America Inc.). Each organoid image was split into ten regions of interest (ROI) to measure the color intensity value. To avoid bias, whole organoids were evaluated since fluorescence was not uniform throughout the specimens. Student’s *t*-test was used to perform pairwise comparisons of the fluorescence of non-PD organoids and PD organoids.

## 5. Conclusions

The data show that neurophysiology is dramatically different between a non-PD and PD organoids and the response to viral infection is altered in PD organoids. This is of significant concern given the rising numbers of persons with neurodegenerative/neurological disease. Could viral infection with a neurotropic virus cause or exacerbate the development of neurological disease in persons predisposed for such conditions? The differential antiviral response of PD organoids highlights the need for more research in neurotropic infections in a neurologically compromised host.

## Figures and Tables

**Table 1:** Validation of gene expression, immunofluorescence data. RT-PCR was performed on RNA obtained from 12 pooled organoids from each cell line 14 days p.i. Data represent the average Ct of 3 replicate RT-PCR reactions. Pairwise comparisons of Ct values were performed via Student’s *t*-test to identify differences in expression. Where available, ∆∆Ct values were obtained from Tables S1 and S2.

	Non-PD	Non-PD + CHIKV	∆∆Ct	PD	PD + CHIKV	∆∆Ct
Marker	AVG Ct ± SE	AVG Ct ± SE		AVG Ct ± SE	AVG Ct ± SE	
GFAP	29.7 ± 0.2	28.1 ± 0.5		29.4 ± 0.5	32.1 ± 0.6	
Iba1	26.2 ± 0.05	26.4 ± 0.03		24.2 ± 0.01	27.9 ± 0.01	
CCR5	31.3 ± 0.01	26.2 ± 0.1	−3.403	30.1 ± 0.2	35.7 ± 1.3	2.824
DRD1	27.6 ± 0.2	27.0 ± 0.7		28.5 ± 0.07	30.5 ± 0.1	
TH	25.2 ± 0.1	28.5 ± 0.2		27.6 ± 0.2	30.2 ± 0.5	
CRYM	25.6 ± 0.01	26.1 ± 0.0		27.9 ± 0.04	31.4 ± 0.0	
GBA	22 ± 0.06	24.4 ± 0.02		25.1 ± 0.1	26.6 ± 0.03	
Aldh1l1	21.4 ± 0.1	20.9 ± 0.2		21.5 ± 0.1	25.6 ± 0.4	
IL-1a	23.4 ± 0.1	22.1 ± 0.1	−11.452	26.1 ± 0.1	27.7 ± 0.2	6.585
IL-10	30.9 ± 0.1	25.0 ± 0.1	8.618	29.6 ± 0.3	33.9 ± 0.9	−2.733
CXCL10	24.5 ± 0.1	21.4 ± 0.1	−2.368	26.6 ± 0.2	23.4 ± 0.02	−4.98
HTR3B	29.5 ± 0.1	25.9 ± 0.1	−6.522	29.3 ± 0.1	34.2 ± 1.3	0.944
SLC6A4	24.9 ± 0.01	24.3 ± 0.04	13.521	28.8 ± 0.5	32.7 ± 0.7	−3.885

**Table 2:** Primers used for detection of specific genes. Primers were designed in Primerquest (IDT SciTools) from human transcripts obtained from NCBI Nucleotide. Ascension numbers were obtained from NCBI Nucleotide database.

Gene	Accension #	Forward Primer 5′-3′	Reverse Primer 5′-3′
GFAP	NG_008401	TACCCTTCTCTGTTTGCTGTG	CCTCCCAAAGTGCTAGGATTAC
Iba1	D86438	CTGAAACGAATGCTGGAGAAAC	GAGAAAGTCAGGGTAGCTGAAC
CCR5	AY463215	CCCAGTGGGACTTTGGAAATA	CGATTGTCAGGAGGATGATGAA
DRD1	NM_000794	AGGGACTTCTCTGTTCGTATCC	GGAACCTGATAACGGCAGCA
TH	BC143611.1	TCATCACCTGGTCACCAAGTT	GGTCGCCGTGCCTGTACT
CRYM	BC018061	GAGCTGGGAGAAGTGATTAAGG	TGGCTGCAACTGTGTCTT
GBA	AH006907	GCTACTCCATTCACACCTACC	GAGCTGACTCTGTCCCTTTAAT
SLC6A4	NM_001045	TGCCCTCTCTTGCAGAATAAC	ATCACCTCCGAGCTCTCTATC
HTR3B	NG_011483	GTCCTGAGTTTGGAGGTAGTTG	CTGAAGGAGATGCCTGAGATAC
IL-1a	BC013142	CTGAAGGAGATGCCTGAGATAC	GATGGGCAACTGATGTGAAATAG
CXCL10	BC010954	CCATTCTGATTTGCTGCCTTATC	TACTAATGCTGATGCAGGTACAG
IL-10	Z30175	CACACACACACACACACAAATC	CTGGATAGGAGGTCCCTTACTT
Aldh1l1	BC027241	GTCAACCAGCAGAGCAAAC	GGCCCATAACCAGGAACAATA

**Table 3:** Antibodies used for immunofluorescence studies.

Antibody	Host	Type	Source	Dilution
**Primary Antibodies**				
Syntaxin 1A	Mouse	Monoclonal	Novus Biologicals	1:1000
Syntaxin 3	Rabbit	Polyclonal	Novus Biologicals	1:500
CYP46A1	Rabbit	Polyclonal	Invitrogen	1:500
ICAM-1/CD54	Mouse	Monoclonal	Novus Biologicals	1:1000
SELE/CD62E	Mouse	Monoclonal	Novus Biologicals	1:1000
CCR7	Mouse	Monoclonal	Novus Biologicals	1:1000
NMDAR2C	Rabbit	Polyclonal	Novus Biologicals	1:1000
MAP2	Chicken	Polyclonal	Novus Biologicals	1:5000
NMDAR1	Mouse	Monoclonal	Novus Biological	1:1000
Synapsin 1	Rabbit	Polyclonal	Invitrogen	1:1000
SOX2	Mouse	Polyclonal	EMD Millipore	1:1000
Tuj1	Rabbit	Monoclonal	EMD Millipore	1:1000
Neurofilament	Mouse	Monoclonal	EMD Millipore	1:1000
Ubiquilin	Rabbit	Polyclonal	Novus Biologicals	1:1000
Capsase 3	Rabbit	Polyclonal	Novus Biologicals	1:1000
GFAP	Rabbit	Polyclonal	Novus Biologicals	1:1000
CHIKV CHK-48	Mouse	Monoclonal	BEI Resources	1:1000
**Secondary Antibodies**				
Anti-Chicken	Goat	Alexa fluor 488	Novus Biologicals	1:3000
Anti-Rabbit	Goat	Alexa fluor 594	Novus Biologicals	1:1000
Anti-Mouse	Goat	Alexa fluor 647	Novus Biologicals	1:2000

## Data Availability

All data are contained in the manuscript.

## Supplementary Materials

The following are available online at https://www.mdpi.com/article/10.3390/pathogens10070913/s1, Table S1: Gene expression data and analysis for non-PD organoids and non-PD organoids infected with CHIKV obtained 14 days post inoculation. UDT, undetermined; Table S2. Gene expression data and analysis for PD organoids and PD organoids infected with CHIKV obtained 14 days post inoculation. UDT, undetermined; Table S3. Gene expression data and analysis for non-PD organoids and PD organoids infected with CHIKV obtained 14 days post inoculation. UDT, undetermined.
