# Supplementary material for: Cerebral Organoids Derived from a Parkinson’s Patient Exhibit Unique Pathogenesis from Chikungunya Virus Infection When Compared to a Non-Parkinson’s Patient
Source: Pathogens. 2021 Jul 20;10(7):913. doi: 10.3390/pathogens10070913 (PMC8308834; doi:10.3390/pathogens10070913)
Supplement: Supplementary file 1 [file pathogens-10-00913-s001.zip › Supplementary Tables.pdf]

Table S1. Gene expression data and analysis for non−PD organoids and non−PD organoids infected with CHIKV obtained 14 days post inoculation. UDT: undetermined

	**Non−PD**	**DCt**	**non−PD−CHIKV**	**DCt**	**DDCt**	**fold change**	**log fold change**
Gene ID	raw CT		raw CT				
18S	4.024		24.588				
GUSB	22.349		25.140				
HPRT1	21.835		30.495				
GAPDH	16.749		31.949				
ABAT	22.019	0.330	27.031	−1.891	2.221	0.215	−2.221
ACHE	25.873	−3.523	34.739	−9.599	6.075	0.015	−6.075
BSN	27.771	−5.421	34.371	−9.231	3.809	0.071	−3.809
CHAT	31.140	−8.790	35.035	−9.894	1.104	0.465	−1.104
CHRM1	30.327	−7.978	32.839	−7.699	−0.279	1.214	0.279
CHRM2	26.637	−4.287	29.489	−4.348	0.061	0.959	−0.061
CHRM3	24.891	−2.541	32.225	−7.085	4.543	0.043	−4.543
CHRM4	25.924	−3.574	29.612	−4.472	0.898	0.537	−0.898
CHRM5	28.195	−5.845	33.284	−8.144	2.299	0.203	−2.299
CHRNA2	31.187	−8.838	36.352	−11.212	2.374	0.193	−2.374
CHRNA3	27.334	−4.985	33.317	−8.177	3.192	0.109	−3.192
CHRNA4	28.765	−6.416	32.343	−7.202	0.786	0.580	−0.786
CHRNA5	27.223	−4.874	32.583	−7.443	2.569	0.169	−2.569
CHRNA6	28.147	−5.797	35.831	−10.691	4.894	0.034	−4.894
CHRNB2	27.406	−5.056	31.301	−6.161	1.104	0.465	−1.104
CHRNB3	28.456	−6.107	34.776	−9.636	3.530	0.087	−3.530
CHRNB4	25.612	−3.263	29.826	−4.686	1.423	0.373	−1.423
CHRND	30.959	−8.610	41.000	−15.860	7.250	0.007	−7.250
CHRNE	28.223	−5.874	41.000	−15.860	9.986	0.001	−9.986
COMT	21.685	0.664	32.018	−6.878	7.542	0.005	−7.542
CPLX1	27.547	−5.198	35.586	−10.446	5.248	0.026	−5.248
DRD2	25.649	−3.300	32.949	−7.809	4.509	0.044	−4.509
DRD3	36.286	−13.937	41.000	−15.860	1.923	0.264	−1.923
GABBR1	22.526	−0.177	25.539	−0.399	0.222	0.857	−0.222
GABRA1	29.916	−7.566	32.633	−7.493	−0.074	1.052	0.074
GABRA2	33.256	−10.907	41.000	−15.860	4.953	0.032	−4.953
GABRA4	33.197	−10.847	41.000	−15.860	5.012	0.031	−5.012
GABRA5	28.907	−6.558	31.817	−6.677	0.119	0.921	−0.119

GABRA6	36.795	−14.44<br>6	37.234	−12.093	−2.352	5.107	2.352
GABRB1	27.857	−5.507	33.106	−7.965	2.458	0.182	−2.458
GABRB2	26.008	−3.659	30.907	−5.767	2.108	0.232	−2.108
GABRB3	24.540	−2.190	30.370	−5.229	3.039	0.122	−3.039
GABRD	29.867	−7.518	41.000	−15.860	8.342	0.003	−8.342
GABRE	26.860	−4.511	31.874	−6.734	2.222	0.214	−2.222
GABRG1	31.466	−9.117	33.935	−8.795	−0.322	1.250	0.322
GABRG2	27.734	−5.385	29.373	−4.233	−1.153	2.223	1.153
GABRG3	29.898	−7.548	31.249	−6.109	−1.439	2.712	1.439
GABRP	19.753	2.596	35.749	−10.609	13.205	0.000	−13.205
GABRQ	26.148	−3.799	32.232	−7.092	3.293	0.102	−3.293
GABRR1	31.249	−8.899	41.000	−15.860	6.960	0.008	−6.960
GABRR2	28.487	−6.138	41.000	−15.860	9.722	0.001	−9.722
GAD1	26.516	−4.167	27.882	−2.742	−1.425	2.686	1.425
GCH1	27.301	−4.952	35.797	−10.656	5.704	0.019	−5.704
GLRA1	29.351	−7.001	33.316	−8.175	1.174	0.443	−1.174
GLRA2	29.577	−7.228	31.837	−6.697	−0.531	1.445	0.531
GLRA3	31.454	−9.104	32.149	−7.009	−2.095	4.273	2.095
GLRB	25.426	−3.077	30.637	−5.497	2.420	0.187	−2.420
GRIA1	27.155	−4.806	27.578	−2.438	−2.368	5.162	2.368
GRIK4	30.909	−8.560	34.203	−9.063	0.503	0.706	−0.503
HTR1B	25.922	−3.572	30.177	−5.036	1.464	0.363	−1.464
HTR1E	25.996	−3.646	31.765	−6.624	2.978	0.127	−2.978
HTR2A	27.635	−5.286	32.851	−7.711	2.425	0.186	−2.425
HTR3A	32.637	−10.28<br>8	35.926	−10.786	0.498	0.708	−0.498
HTR3B	39.939	−17.59<br>0	36.209	−11.068	−6.522	91.881	6.522
HTR7	28.161	−5.811	41.000	−15.860	10.048	0.001	−10.048
MAOA	20.656	1.694	34.164	−9.024	10.718	0.001	−10.718
PHOX2A	29.938	−7.588	41.000	−15.860	8.271	0.003	−8.271
RIMS1	29.683	−7.334	37.947	−12.807	5.473	0.023	−5.473
RIMS3	27.063	−4.713	31.775	−6.635	1.921	0.264	−1.921
RIMS4	24.171	−1.822	28.836	−3.695	1.873	0.273	−1.873
SLC18A2	27.918	−5.569	35.930	−10.790	5.221	0.027	−5.221
SLC18A3	28.925	−6.576	31.935	−6.795	0.219	0.859	−0.219
SLC1A3	22.752	−0.402	25.788	−0.648	0.246	0.843	−0.246
SLC32A1	32.869	−10.52<br>0	32.586	−7.446	−3.075	8.424	3.075
SLC5A7	26.900	−4.550	32.400	−7.260	2.710	0.153	−2.710

SLC6A1	29.817	−7.468	32.352	−7.212	−0.256	1.194	0.256
SLC6A11	25.801	−3.452	31.115	−5.975	2.523	0.174	−2.523
SLC6A12	27.546	−5.197	35.466	−10.326	5.129	0.029	−5.129
SLC6A13	31.415	−9.066	41.000	−15.860	6.794	0.009	−6.794
SLC6A15	25.256	−2.907	30.701	−5.561	2.654	0.159	−2.654
SLC6A16	37.044	−14.695	36.309	−11.169	−3.526	11.517	3.526
SLC6A18	35.747	−13.398	41.000	−15.860	2.462	0.182	−2.462
SLC6A2	29.022	−6.672	34.032	−8.891	2.219	0.215	−2.219
SLC6A20	28.239	−5.890	33.795	−8.654	2.765	0.147	−2.765
SLC6A4	24.688	−2.339	41.000	−15.860	13.521	0.000	−13.521
SLC6A5	29.312	−6.963	31.365	−6.225	−0.738	1.668	0.738
SLC6A6	24.076	−1.726	30.917	−5.777	4.050	0.060	−4.050
SLC6A7	33.942	−11.593	34.456	−9.316	−2.277	4.848	2.277
SLC6A8	21.614	0.736	29.670	−4.530	5.265	0.026	−5.265
SLC6A9	28.075	−5.726	41.000	−15.860	10.134	0.001	−10.134
SNPH	24.745	−2.396	41.000	−15.860	13.464	0.000	−13.464
STX1A	25.476	−3.126	34.270	−9.129	6.003	0.016	−6.003
STX1B	24.932	−2.582	29.870	−4.730	2.148	0.226	−2.148
STX3	23.130	−0.781	34.481	−9.341	8.560	0.003	−8.560
STXBP1	22.473	−0.124	28.751	−3.611	3.487	0.089	−3.487
SV2A	22.870	−0.520	29.740	−4.600	4.080	0.059	−4.080
SYN1	25.416	−3.067	29.018	−3.878	0.811	0.570	−0.811
SYN2	24.842	−2.493	29.858	−4.717	2.225	0.214	−2.225
SYN3	25.811	−3.462	29.486	−4.346	0.884	0.542	−0.884
SYP	23.301	−0.951	27.844	−2.704	1.753	0.297	−1.753
SYT1	24.036	−1.687	28.535	−3.394	1.708	0.306	−1.708
TPH1	21.969	0.380	33.496	−8.356	8.736	0.002	−8.736
18S	4.418						
GUSB	23.498		26.753				
HPRT1	23.007	0.492	31.987	−5.234	5.726	0.019	−5.726
GAPDH	18.410		31.307	−4.554	4.554	0.043	−4.554
IL1A	26.294	−2.795	41.000	−14.247	11.452	0.000	−11.452
IL1B	25.902	−2.404	37.074	−10.321	7.917	0.004	−7.917
IL2	37.521	−14.022	41.000	−14.247	0.225	0.856	−0.225
IL3	UDT		UDT				
IL4	UDT		UDT				
IL5	36.911	−13.413	41.000	−14.247	0.834	0.561	−0.834

IL6	28.923	−5.425	36.788	−10.035	4.610	0.041	−4.610
IL7	29.994	−6.496	36.807	−10.054	3.558	0.085	−3.558
IL8	23.324	0.174	31.975	−5.222	5.396	0.024	−5.396
IL9	36.586	−13.088	41.000	−14.247	1.159	0.448	−1.159
IL10	29.127	−5.629	41.000	−14.247	8.618	0.003	−8.618
IL12A	31.476	−7.977	34.297	−7.544	−0.433	1.350	0.433
IL12B	36.211	−12.713	41.000	−14.247	1.534	0.345	−1.534
IL13	37.285	−13.786	41.000	−14.247	0.461	0.727	−0.461
IL15	31.824	−8.326	41.000	−14.247	5.921	0.017	−5.921
IL17A	UDT		UDT				
IL18	30.782	−7.284	41.000	−14.247	6.963	0.008	−6.963
CCL3	31.202	−7.704	36.284	−9.531	1.827	0.282	−1.827
CCL19	30.937	−7.438	33.579	−6.826	−0.612	1.529	0.612
CCL2	22.749	0.750	30.598	−3.845	4.595	0.041	−4.595
CCL5	29.127	−5.629	28.539	−1.786	−3.843	14.346	3.843
CCR2	38.102	−14.604	41.000	−14.247	−0.357	1.281	0.357
CCR4	30.485	−6.987	30.483	−3.730	−3.257	9.557	3.257
CCR5	34.342	−10.844	41.000	−14.247	3.403	0.095	−3.403
CCR7	29.918	−6.420	41.000	−14.247	7.827	0.004	−7.827
CXCR3	34.716	−11.217	41.000	−14.247	3.030	0.122	−3.030
CXCL10	27.561	−4.063	28.448	−1.695	−2.368	5.162	2.368
CXCL11	28.559	−5.060	28.639	−1.886	−3.174	9.026	3.174
CSF1	23.859	−0.360	30.444	−3.691	3.331	0.099	−3.331
CSF2	30.007	−6.508	41.000	−14.247	7.739	0.005	−7.739
CSF3	28.321	−4.823	41.000	−14.247	9.424	0.001	−9.424
STAT3	21.286	2.213	27.303	−0.550	2.763	0.147	−2.763
NFKB2	24.020	−0.521	30.886	−4.133	3.612	0.082	−3.612
IKBKB	24.590	−1.092	33.643	−6.890	5.798	0.018	−5.798
CD3E	32.383	−8.884	41.000	−14.247	5.363	0.024	−5.363
CD4	28.869	−5.370	34.384	−7.631	2.261	0.209	−2.261
CD8A	28.828	−5.330	30.385	−3.632	−1.698	3.244	1.698
CD19	36.392	−12.893	41.000	−14.247	1.354	0.391	−1.354
IL2RA	34.444	−10.946	41.000	−14.247	3.301	0.101	−3.301

CD28	34.856	−11.358	41.000	−14.247	2.889	0.135	−2.889
CD38	32.191	−8.693	33.777	−7.024	−1.669	3.180	1.669
CD40	26.560	−3.062	34.174	−7.421	4.359	0.049	−4.359
PTPRC	32.506	−9.008	37.057	−10.304	1.296	0.407	−1.296
CD68	23.904	−0.405	29.936	−3.183	2.778	0.146	−2.778
CD80	33.718	−10.220	41.000	−14.247	4.027	0.061	−4.027
CD86	34.604	−11.106	41.000	−14.247	3.141	0.113	−3.141
CTLA4	34.564	−11.065	41.000	−14.247	3.182	0.110	−3.182
CD40LG	39.231	−15.732	41.000	−14.247	−1.485	2.800	1.485
HLA−DRA	30.344	−6.846	34.872	−8.119	1.273	0.414	−1.273
HLA−DRB1	UDT		UDT				
TBX21	31.993	−8.495	41.000	−14.247	5.752	0.019	−5.752
TNFRSF18	30.559	−7.060	37.496	−10.743	3.683	0.078	−3.683
ICOS	36.160	−12.662	41.000	−14.247	1.585	0.333	−1.585
NOS2	31.609	−8.111	33.575	−6.822	−1.289	2.443	1.289
BCL2	24.410	−0.912	29.992	−3.239	2.327	0.199	−2.327
BCL2L1	26.495	−2.997	34.109	−7.356	4.359	0.049	−4.359
BAX	21.558	1.940	28.503	−1.750	3.690	0.077	−3.690
ICAM1	24.207	−0.708	32.995	−6.242	5.534	0.022	−5.534
SELP	29.187	−5.689	41.000	−14.247	8.558	0.003	−8.558
SELE	28.415	−4.916	41.000	−14.247	9.331	0.002	−9.331
HMOX1	23.861	−0.363	32.930	−6.177	5.814	0.018	−5.814
PTGS2	24.462	−0.964	33.354	−6.601	5.637	0.020	−5.637
LRP2	30.028	−6.529	31.890	−5.137	−1.392	2.625	1.392
CYP1A2	UDT		UDT				
CYP7A1	33.329	−9.830	41.000	−14.247	4.417	0.047	−4.417
IFNG	UDT		UDT				
PRF1	34.518	−11.020	41.000	−14.247	3.227	0.107	−3.227
GZMB	36.465	−12.967	41.000	−14.247	1.280	0.412	−1.280
GNLY	UDT		UDT				
FAS	24.398	−0.899	33.822	−7.069	6.170	0.014	−6.170
FASLG	UDT		UDT				

TGFB1	22.243	1.256	29.953	−3.200	4.456	0.046	−4.456
SMAD3	23.706	−0.207	30.837	−4.084	3.877	0.068	−3.877
SMAD7	24.199	−0.701	32.804	−6.051	5.350	0.025	−5.350
SKI	21.226	2.272	27.095	−0.342	2.614	0.163	−2.614
FN1	16.018	7.481	26.893	−0.140	7.621	0.005	−7.621
C3	21.407	2.091	31.205	−4.452	6.543	0.011	−6.543
TNF	29.850	−6.352	35.580	−8.827	2.475	0.180	−2.475
LTA	33.964	−10.465	36.279	−9.526	−0.939	1.917	0.939
ACE	28.256	−4.757	34.543	−7.790	3.033	0.122	−3.033
VEGFA	23.275	0.224	28.965	−2.212	2.436	0.185	−2.436
CD34	28.925	−5.426	41.000	−14.247	8.821	0.002	−8.821
AGTR1	32.417	−8.918	41.000	−14.247	5.329	0.025	−5.329
AGTR2	30.021	−6.522	36.485	−9.732	3.210	0.108	−3.210
EDN1	23.585	−0.087	33.951	−7.198	7.111	0.007	−7.111
LIF	23.354	0.145	33.222	−6.469	6.614	0.010	−6.614
LY96	28.819	−5.320	39.630	−12.877	7.557	0.005	−7.557
MIF	18.354	5.144	23.188	3.565	1.579	0.335	−1.579
NFATC3	23.310	0.188	26.618	0.135	0.053	0.964	−0.053
NFATC4	23.702	−0.204	31.321	−4.568	4.364	0.049	−4.364
PF4	32.745	−9.246	41.000	−14.247	5.001	0.031	−5.001
SYK	25.367	−1.869	33.654	−6.901	5.032	0.031	−5.032
18S	4.975		19.344				
GUSB	24.178		26.217				
HPRT1	23.756	0.422	31.480	−5.263	5.685	0.019	−5.685
GAPDH	18.910	5.268	30.780	−4.563	9.831	0.001	−9.831
ABCA1	22.968	1.211	32.539	−6.322	7.533	0.005	−7.533
ADAM10	21.874	2.304	29.774	−3.557	5.861	0.017	−5.861
ADAM17	24.215	−0.037	31.301	−5.084	5.047	0.030	−5.047
ADAM9	20.640	3.538	27.340	−1.123	4.661	0.040	−4.661
APBA1	26.612	−2.434	29.538	−3.321	0.888	0.540	−0.888
APBA2	27.247	−3.069	31.126	−4.909	1.840	0.279	−1.840
APBA3	26.384	−2.205	33.519	−7.302	5.096	0.029	−5.096
APBB1	24.973	−0.795	29.453	−3.236	2.441	0.184	−2.441
APBB2	23.635	0.543	30.642	−4.425	4.968	0.032	−4.968
APBB3	26.463	−2.284	34.929	−8.712	6.428	0.012	−6.428
APCS	UDT		UDT				
APH1A	25.096	−0.918	29.971	−3.754	2.836	0.140	−2.836
APH1B	24.753	−0.575	30.766	−4.549	3.975	0.064	−3.975
APLP1	26.146	−1.968	29.696	−3.479	1.511	0.351	−1.511
APLP2	21.738	2.440	29.563	−3.346	5.787	0.018	−5.787

APOE	20.729	3.449	29.871	−3.654	7.103	0.007	−7.103
APP	18.600	5.578	23.331	2.886	2.692	0.155	−2.692
BACE1	24.263	−0.085	30.446	−4.229	4.144	0.057	−4.144
BACE2	22.657	1.521	28.379	−2.162	3.683	0.078	−3.683
CAPN1	21.127	3.051	30.895	−4.678	7.729	0.005	−7.729
CASP3	25.482	−1.304	32.375	−6.158	4.854	0.035	−4.854
CASP6	24.998	−0.819	31.753	−5.536	4.716	0.038	−4.716
CDC2	25.776	−1.598	31.789	−5.572	3.975	0.064	−3.975
CDK5	25.157	−0.978	29.955	−3.738	2.760	0.148	−2.760
CDK5R1	25.552	−1.374	27.194	−0.977	−0.397	1.317	0.397
SLC18A3	30.740	−6.562	33.265	−7.048	0.486	0.714	−0.486
CSNK1A1	24.199	−0.021	30.176	−3.959	3.938	0.065	−3.938
CTSB	20.521	3.658	29.549	−3.332	6.989	0.008	−6.989
CTSC	19.266	4.912	29.442	−3.225	8.137	0.004	−8.137
CTSD	20.005	4.173	30.636	−4.419	8.592	0.003	−8.592
CTSG	UDT		UDT				
BPTF	23.545	0.633	28.645	−2.428	3.061	0.120	−3.061
GJB1	29.543	−5.364	30.458	−4.241	−1.123	2.178	1.123
GLS	23.467	0.711	32.707	−6.490	7.202	0.007	−7.202
GRIN1	27.845	−3.666	25.970	0.247	−3.914	15.070	3.914
GRIN2A	28.442	−4.263	33.627	−7.411	3.147	0.113	−3.147
GRIN2B	30.698	−6.520	29.232	−3.015	−3.504	11.349	3.504
GRIN2D	24.497	−0.319	30.824	−4.607	4.289	0.051	−4.289
GSK3B	23.268	0.910	26.007	0.210	0.700	0.616	−0.700
HSD17B10	23.409	0.769	29.477	−3.260	4.029	0.061	−4.029
IDE	25.395	−1.216	31.278	−5.062	3.845	0.070	−3.845
IFNG	UDT		UDT				
IL1A	26.896	−2.718	35.520	−9.303	6.585	0.010	−6.585
IL1B	26.574	−2.396	UDT				
IL6	29.742	−5.563	37.171	−10.954	5.391	0.024	−5.391
INS	36.081	−11.902	41.000	−14.783	2.881	0.136	−2.881
INSR	24.664	−0.486	33.270	−7.053	6.567	0.011	−6.567
LRP1	20.966	3.212	29.703	−3.486	6.699	0.010	−6.699
LRP2	30.842	−6.664	32.670	−6.453	−0.211	1.158	0.211
LRPAP1	22.717	1.461	30.572	−4.355	5.817	0.018	−5.817
MAPK1	24.405	−0.227	28.598	−2.381	2.154	0.225	−2.154
MAPK3	23.677	0.501	28.830	−2.613	3.115	0.115	−3.115
MAPT	24.924	−0.746	24.552	1.665	−2.411	5.318	2.411
MME	23.724	0.455	36.304	−10.087	10.542	0.001	−10.542
NCSTN	23.617	0.561	30.541	−4.325	4.886	0.034	−4.886

PDE8B	25.386	−1.208	34.360	−8.143	6.935	0.008	−6.935
PSENEN	31.480	−7.302	32.922	−6.705	−0.597	1.512	0.597
PLD1	24.803	−0.624	34.716	−8.499	7.875	0.004	−7.875
PPP2CA	22.875	1.304	29.710	−3.493	4.796	0.036	−4.796
PRKACB	25.885	−1.707	29.953	−3.736	2.029	0.245	−2.029
PRKCA	27.197	−3.018	31.386	−5.169	2.151	0.225	−2.151
PRKCB	25.911	−1.733	28.649	−2.432	0.700	0.616	−0.700
PRKCE	27.441	−3.263	31.072	−4.855	1.592	0.332	−1.592
PRKCG	30.863	−6.685	32.720	−6.503	−0.182	1.135	0.182
PKN1	22.940	1.238	28.526	−2.309	3.547	0.086	−3.547
PSEN1	24.909	−0.731	34.378	−8.161	7.430	0.006	−7.430
PSEN2	26.583	−2.405	35.637	−9.420	7.015	0.008	−7.015
SERPINA3	26.133	−1.955	32.726	−6.509	4.554	0.043	−4.554
SNCA	23.510	0.669	29.709	−3.492	4.160	0.056	−4.160
SOAT1	25.510	−1.332	33.852	−7.635	6.303	0.013	−6.303
SOD2	21.755	2.423	29.323	−3.106	5.529	0.022	−5.529
CAPNS2	23.103	1.075	29.752	−3.535	4.610	0.041	−4.610
TNF	30.548	−6.370	41.000	−14.783	8.414	0.003	−8.414
UCHL1	21.707	2.471	26.617	−0.400	2.871	0.137	−2.871
VSNL1	27.649	−3.471	34.690	−8.473	5.002	0.031	−5.002
GAL	29.142	−4.964	33.295	−7.078	2.114	0.231	−2.114
ACHE	24.642	−0.464	35.310	−9.093	8.629	0.003	−8.629
AGER	27.959	−3.780	41.000	−14.783	11.003	0.000	−11.003
NAE1	26.691	−2.513	33.495	−7.278	4.766	0.037	−4.766
BCHE	28.987	−4.809	38.093	−11.876	7.068	0.007	−7.068
CAPNS1	20.366	3.812	29.737	−3.521	7.333	0.006	−7.333
CHRNA7	28.672	−4.493	34.965	−8.748	4.255	0.052	−4.255
CSNK1D	21.767	2.411	28.475	−2.258	4.669	0.039	−4.669
CYP46A1	35.514	−11.335	41.000	−14.783	3.448	0.092	−3.448
GAP43	24.747	−0.568	29.559	−3.342	2.774	0.146	−2.774
GRIN2C	27.815	−3.637	36.335	−10.118	6.481	0.011	−6.481
SLC30A3	33.262	−9.083	37.525	−11.308	2.225	0.214	−2.225
ST6GAL1	28.664	−4.486	33.207	−6.990	2.504	0.176	−2.504
UBQLN1	22.741	1.438	28.774	−2.557	3.995	0.063	−3.995

Table S2. Gene expression data and analysis for PD organoids and PD organoids infected with CHIKV obtained 14 days post inoculation. UDT: undetermined

	PD	DCt	PD–CHI KV	DCt	DDCt	fold change	log fold change
Gene ID	raw CT						
18S	16.131		16.565				
GUSB	28.835		27.891				
HPRT1	29.402		28.859				
GAPDH	23.696		23.526				
ABAT	26.914	1.920	27.652	0.239	1.682	0.312	−1.682
ACHE	33.431	−4.596	32.699	−4.808	0.212	0.863	−0.212
BSN	32.680	−3.845	33.529	−5.639	1.794	0.288	−1.794
CHAT	33.163	−4.328	32.772	−4.881	0.554	0.681	−0.554
CHRM1	34.386	−5.551	35.210	−7.319	1.768	0.294	−1.768
CHRM2	28.788	0.046	30.496	−2.605	2.652	0.159	−2.652
CHRM3	30.742	−1.907	33.300	−5.409	3.502	0.088	−3.502
CHRM4	28.544	0.291	32.125	−4.234	4.525	0.043	−4.525
CHRM5	33.593	−4.758	33.617	−5.727	0.968	0.511	−0.968
CHRNA2	33.707	−4.872	33.569	−5.679	0.806	0.572	−0.806
CHRNA3	33.995	−5.160	31.535	−3.644	−1.515	2.859	1.515
CHRNA4	30.011	−1.176	30.347	−2.457	1.281	0.412	−1.281
CHRNA5	33.637	−4.802	32.700	−4.809	0.007	0.995	−0.007
CHRNA6	34.543	−5.708	33.552	−5.662	−0.047	1.033	0.047
CHRNB2	30.461	−1.626	30.712	−2.821	1.195	0.437	−1.195
CHRNB3	34.004	−5.169	34.036	−6.145	0.976	0.508	−0.976
CHRNB4	30.293	−1.458	29.375	−1.485	0.027	0.982	−0.027
CHRND	33.508	−4.673	31.779	−3.889	−0.784	1.722	0.784
CHRNE	35.241	−6.406	36.525	−8.635	2.229	0.213	−2.229
COMT	30.211	−1.376	32.470	−4.579	3.203	0.109	−3.203
CPLX1	32.251	−3.417	32.332	−4.441	1.025	0.492	−1.025
DRD2	30.605	−1.770	31.010	−3.119	1.349	0.393	−1.349
DRD3	38.139	−9.304	41.000	−13.109	3.805	0.072	−3.805
GABBR1	25.598	3.237	25.862	2.029	1.208	0.433	−1.208
GABRA1	32.755	−3.920	34.206	−6.315	2.395	0.190	−2.395
GABRA2	34.440	−5.605	35.238	−7.348	1.743	0.299	−1.743
GABRA4	36.282	−7.447	35.484	−7.593	0.146	0.904	−0.146
GABRA5	28.163	0.672	30.221	−2.330	3.001	0.125	−3.001
GABRA6	41.000	−12.165	41.000	−13.109	0.944	0.520	−0.944
GABRB1	29.976	−1.141	32.454	−4.563	3.422	0.093	−3.422
GABRB2	32.442	−3.607	34.744	−6.853	3.246	0.105	−3.246
GABRB3	26.651	2.184	30.497	−2.606	4.791	0.036	−4.791
GABRD	35.787	−6.952	35.923	−8.033	1.081	0.473	−1.081
GABRE	31.348	−2.513	30.511	−2.620	0.107	0.928	−0.107

GABRG1	35.851	−7.016	34.730	−6.840	−0.176	1.130	0.176
GABRG2	29.925	−1.090	30.851	−2.960	1.870	0.274	−1.870
GABRG3	32.351	−3.516	33.147	−5.256	1.740	0.299	−1.740
GABRP	34.678	−5.843	34.391	−6.501	0.658	0.634	−0.658
GABRQ	28.760	0.075	29.819	−1.928	2.004	0.249	−2.004
GABRR1	34.513	−5.678	36.634	−8.743	3.065	0.119	−3.065
GABRR2	34.542	−5.707	34.463	−6.572	0.865	0.549	−0.865
GAD1	31.556	−2.721	31.970	−4.080	1.359	0.390	−1.359
GCH1	34.512	−5.677	32.409	−4.518	−1.159	2.232	1.159
GLRA1	28.739	0.096	30.983	−3.093	3.188	0.110	−3.188
GLRA2	29.496	−0.661	27.889	0.002	−0.663	1.583	0.663
GLRA3	31.541	−2.706	31.165	−3.274	0.569	0.674	−0.569
GLRB	29.934	−1.099	29.358	−1.467	0.368	0.775	−0.368
GRIA1	26.972	1.863	27.188	0.703	1.160	0.447	−1.160
GRIK4	33.695	−4.861	33.486	−5.595	0.734	0.601	−0.734
HTR1B	30.941	−2.106	31.236	−3.345	1.240	0.423	−1.240
HTR1E	32.254	−3.419	31.614	−3.723	0.305	0.810	−0.305
HTR2A	29.439	−0.604	29.740	−1.849	1.246	0.422	−1.246
HTR3A	34.370	−5.535	31.314	−3.423	−2.112	4.322	2.112
HTR3B	41.000	−12.165	41.000	−13.109	0.944	0.520	−0.944
HTR7	33.555	−4.720	36.188	−8.298	3.577	0.084	−3.577
MAOA	27.679	1.156	28.404	−0.513	1.669	0.315	−1.669
PHOX2A	34.196	−5.361	31.973	−4.082	−1.278	2.426	1.278
RIMS1	34.754	−5.919	34.553	−6.663	0.743	0.597	−0.743
RIMS3	32.461	−3.626	31.719	−3.829	0.202	0.869	−0.202
RIMS4	27.877	0.958	27.578	0.313	0.645	0.639	−0.645
SLC18A2	35.731	−6.896	32.329	−4.438	−2.458	5.495	2.458
SLC18A3	32.810	−3.975	30.856	−2.965	−1.010	2.013	1.010
SLC1A3	23.976	4.859	24.873	3.018	1.842	0.279	−1.842
SLC32A1	37.388	−8.553	41.000	−13.109	4.556	0.043	−4.556
SLC5A7	28.812	0.023	28.373	−0.482	0.505	0.705	−0.505
SLC6A1	30.899	−2.064	32.843	−4.953	2.888	0.135	−2.888
SLC6A11	30.300	−1.465	33.219	−5.328	3.863	0.069	−3.863
SLC6A12	35.825	−6.990	34.648	−6.757	−0.233	1.176	0.233
SLC6A13	37.836	−9.001	37.453	−9.562	0.561	0.678	−0.561
SLC6A15	32.458	−3.623	30.466	−2.575	−1.048	2.067	1.048
SLC6A16	41.000	−12.165	36.867	−8.976	−3.189	9.120	3.189
SLC6A18	41.000	−12.165	41.000	−13.109	0.944	0.520	−0.944
SLC6A2	41.000	−12.165	37.711	−9.821	−2.345	5.079	2.345
SLC6A20	32.169	−3.334	33.407	−5.516	2.182	0.220	−2.182
SLC6A4	41.000	−12.165	36.171	−8.280	−3.885	14.775	3.885

SLC6A5	34.502	−5.667	35.186	−7.295	1.628	0.323	−1.628
SLC6A6	29.683	−0.848	29.763	−1.872	1.024	0.492	−1.024
SLC6A7	32.174	−3.339	41.000	−13.109	9.770	0.001	−9.770
SLC6A8	27.653	1.182	27.658	0.233	0.949	0.518	−0.949
SLC6A9	36.727	−7.892	41.000	−13.109	5.217	0.027	−5.217
SNPH	41.000	−12.165	41.000	−13.109	0.944	0.520	−0.944
STX1A	37.650	−8.815	33.485	−5.594	−3.222	9.328	3.222
STX1B	36.292	−7.457	34.159	−6.268	−1.189	2.281	1.189
STX3	41.000	−12.165	32.384	−4.493	−7.672	203.918	7.672
STXBP1	29.626	−0.791	30.438	−2.547	1.756	0.296	−1.756
SV2A	29.722	−0.887	31.799	−3.909	3.021	0.123	−3.021
SYN1	29.521	−0.686	32.353	−4.462	3.776	0.073	−3.776
SYN2	30.807	−1.972	32.353	−4.462	2.491	0.178	−2.491
SYN3	28.871	−0.036	32.501	−4.611	4.575	0.042	−4.575
SYP	25.757	3.078	30.963	−3.072	6.150	0.014	−6.150
SYT1	28.502	0.333	32.535	−4.644	4.977	0.032	−4.977
TPH1	31.777	−2.942	31.888	−3.997	1.054	0.482	−1.054
18S	14.695		16.386				
GUSB	25.935		29.317				
HPRT1	27.465	−1.530	30.427	−1.110	−0.421	1.339	0.421
GAPDH	21.603	4.332	24.668	4.649	−0.318	1.246	0.318
IL1A	31.130	−5.195	31.867	−2.549	−2.646	6.258	2.646
IL1B	32.355	−6.420	31.447	−2.129	−4.291	19.574	4.291
IL2	41.000	−15.065	41.000	−11.683	−3.383	10.431	3.383
IL3	UDT		UDT				
IL4	UDT		UDT				
IL5	41.000	−15.065	36.810	−7.493	−7.572	190.334	7.572
IL6	32.539	−6.605	31.676	−2.359	−4.246	18.976	4.246
IL7	31.689	−5.754	32.541	−3.223	−2.531	5.780	2.531
IL8	28.216	−2.282	28.371	0.947	−3.228	9.372	3.228
IL9	41.000	−15.065	41.000	−11.683	−3.383	10.431	3.383
IL10	32.505	−6.570	33.155	−3.838	−2.733	6.647	2.733
IL12A	31.978	−6.044	34.378	−5.061	−0.983	1.977	0.983
IL12B	UDT		41.000	−11.683			
IL13	UDT		41.000	−11.683			
IL15	38.156	−12.221	36.154	−6.836	−5.385	41.785	5.385
IL17A	UDT		UDT				
IL18	38.248	−12.314	36.352	−7.035	−5.279	38.825	5.279
CCL3	26.129	−0.194	24.971	4.346	−4.540	23.265	4.540
CCL19	29.821	−3.886	28.579	0.739	−4.625	24.672	4.625
CCL2	26.539	−0.605	25.861	3.456	−4.061	16.689	4.061

CCL5	29.388	−3.453	29.416	−0.099	−3.354	10.228	3.354
CCR2	41.000	−15.065	41.000	−11.683	−3.383	10.431	3.383
CCR4	29.873	−3.939	32.743	−3.425	−0.513	1.427	0.513
CCR5	33.441	−7.507	34.000	−4.682	−2.824	7.084	2.824
CCR7	41.000	−15.065	35.893	−6.576	−8.489	359.385	8.489
CXCR3	41.000	−15.065	41.000	−11.683	−3.383	10.431	3.383
CXCL10	27.483	−1.549	25.886	3.432	−4.980	31.562	4.980
CXCL11	28.931	−2.997	27.641	1.677	−4.674	25.520	4.674
CSF1	26.525	−0.591	27.029	2.289	−2.880	7.359	2.880
CSF2	41.000	−15.065	35.262	−5.945	−9.121	556.700	9.121
CSF3	41.000	−15.065	34.405	−5.088	−9.978	1008.310	9.978
STAT3	24.129	1.806	24.523	4.794	−2.989	7.937	2.989
NFKB2	27.840	−1.906	28.233	1.084	−2.990	7.945	2.990
IKBKB	30.644	−4.710	33.437	−4.120	−0.590	1.506	0.590
CD3E	41.000	−15.065	41.000	−11.683	−3.383	10.431	3.383
CD4	29.730	−3.795	30.220	−0.902	−2.893	7.428	2.893
CD8A	26.917	−0.983	29.450	−0.133	−0.850	1.802	0.850
CD19	41.000	−15.065	41.000	−11.683	−3.383	10.431	3.383
IL2RA	30.241	−4.307	30.578	−1.261	−3.046	8.260	3.046
CD28	31.399	−5.465	30.558	−1.241	−4.224	18.688	4.224
CD38	31.732	−5.797	31.756	−2.439	−3.358	10.257	3.358
CD40	29.393	−3.459	28.813	0.504	−3.963	15.596	3.963
PTPRC	29.796	−3.861	29.612	−0.294	−3.567	11.851	3.567
CD68	24.878	1.056	23.530	5.788	−4.731	26.561	4.731
CD80	41.000	−15.065	34.401	−5.084	−9.982	1011.007	9.982
CD86	32.912	−6.977	32.742	−3.424	−3.553	11.734	3.553
CTLA4	41.000	−15.065	39.481	−10.164	−4.902	29.896	4.902
CD40LG	41.000	−15.065	41.000	−11.683	−3.383	10.431	3.383
HLA−DRA	32.429	−6.495	29.706	−0.389	−6.106	68.864	6.106
HLA−DRB1	UDT		UDT				
TBX21	34.075	−8.141	35.355	−6.038	−2.103	4.296	2.103
TNFRSF18	36.763	−10.829	35.437	−6.119	−4.710	26.165	4.710
ICOS	41.000	−15.065	38.918	−9.601	−5.465	44.157	5.465
NOS2	33.995	−8.061	33.290	−3.973	−4.088	17.002	4.088
BCL2	27.860	−1.925	28.591	0.726	−2.652	6.284	2.652
BCL2L1	30.469	−4.534	30.875	−1.558	−2.976	7.869	2.976
BAX	26.763	−0.828	25.836	3.481	−4.309	19.824	4.309
ICAM1	30.276	−4.341	28.615	0.703	−5.044	32.992	5.044

SELP	41.000	−15.065	35.572	−6.255	−8.811	449.037	8.811
SELE	41.000	−15.065	34.787	−5.470	−9.596	773.646	9.596
HMOX1	29.618	−3.683	28.658	0.660	−4.343	20.291	4.343
PTGS2	31.319	−5.385	30.188	−0.871	−4.514	22.848	4.514
LRP2	29.929	−3.995	30.431	−1.114	−2.881	7.366	2.881
CYP1A2	UDT		UDT				
CYP7A1	41.000	−15.065	41.000	−11.683	−3.383	10.431	3.383
IFNG	UDT		UDT				
PRF1	41.000	−15.065	37.594	−8.277	−6.789	110.566	6.789
GZMB	35.673	−9.738	33.517	−4.200	−5.538	46.472	5.538
GNLY	UDT		UDT				
FAS	30.469	−4.534	30.304	−0.987	−3.547	11.690	3.547
FASLG	UDT		UDT				
TGFB1	26.213	−0.279	24.894	4.423	−4.702	26.030	4.702
SMAD3	28.719	−2.784	28.396	0.921	−3.705	13.043	3.705
SMAD7	28.927	−2.992	28.559	0.758	−3.750	13.453	3.750
SKI	24.155	1.779	25.644	3.673	−1.894	3.717	1.894
FN1	23.783	2.151	22.398	6.919	−4.768	27.247	4.768
C3	28.306	−2.372	25.715	3.602	−5.973	62.833	5.973
TNF	36.932	−10.997	32.780	−3.463	−7.535	185.405	7.535
LTA	36.294	−10.359	35.222	−5.904	−4.455	21.930	4.455
ACE	31.685	−5.750	32.199	−2.882	−2.868	7.303	2.868
VEGFA	26.794	−0.859	28.286	1.031	−1.890	3.707	1.890
CD34	32.341	−6.406	31.857	−2.539	−3.867	14.592	3.867
AGTR1	41.000	−15.065	41.000	−11.683	−3.383	10.431	3.383
AGTR2	33.363	−7.428	30.848	−1.531	−5.897	59.593	5.897
EDN1	30.832	−4.897	29.578	−0.261	−4.636	24.868	4.636
LIF	32.307	−6.372	28.242	1.075	−7.447	174.515	7.447
LY96	33.730	−7.796	32.708	−3.391	−4.405	21.180	4.405
MIF	20.263	5.671	21.299	8.019	−2.347	5.088	2.347
NFATC3	26.453	−0.519	26.465	2.852	−3.371	10.347	3.371
NFATC4	29.433	−3.499	28.665	0.652	−4.151	17.769	4.151
PF4	41.000	−15.065	35.909	−6.591	−8.474	355.584	8.474
SYK	31.194	−5.260	30.376	−1.059	−4.201	18.392	4.201
18S	17.040		13.717				
GUSB	29.611		26.568				
HPRT1	29.974	−0.363	27.704	−1.136	0.773	0.585	−0.773
GAPDH	24.177	5.434	22.247	4.321	1.113	0.462	−1.113
ABCA1	30.755	−1.144	27.724	−1.156	0.012	0.991	−0.012
ADAM10	28.541	1.071	25.861	0.707	0.364	0.777	−0.364
ADAM17	29.957	−0.345	27.438	−0.870	0.524	0.695	−0.524

ADAM9	25.767	3.845	23.335	3.233	0.612	0.654	−0.612
APBA1	28.323	1.289	27.853	−1.285	2.573	0.168	−2.573
APBA2	29.469	0.142	28.970	−2.401	2.544	0.171	−2.544
APBA3	31.592	−1.981	29.356	−2.787	0.807	0.572	−0.807
APBB1	28.080	1.531	26.765	−0.196	1.727	0.302	−1.727
APBB2	28.964	0.648	27.200	−0.632	1.279	0.412	−1.279
APBB3	33.339	−3.728	30.856	−4.288	0.560	0.678	−0.560
APCS	UDT		UDT				
APH1A	28.879	0.732	25.708	0.860	−0.128	1.093	0.128
APH1B	28.414	1.197	26.397	0.171	1.026	0.491	−1.026
APLP1	29.303	0.308	28.392	−1.824	2.132	0.228	−2.132
APLP2	26.288	3.324	25.127	1.442	1.882	0.271	−1.882
APOE	26.450	3.161	24.510	2.059	1.103	0.466	−1.103
APP	21.792	7.820	20.806	5.762	2.058	0.240	−2.058
BACE1	27.974	1.637	26.949	−0.381	2.018	0.247	−2.018
BACE2	25.937	3.675	24.270	2.299	1.376	0.385	−1.376
CAPN1	27.813	1.798	26.721	−0.153	1.951	0.259	−1.951
CASP3	30.874	−1.263	28.933	−2.365	1.103	0.466	−1.103
CASP6	30.816	−1.204	27.531	−0.963	−0.242	1.182	0.242
CDC2	31.796	−2.185	28.746	−2.178	−0.007	1.005	0.007
CDK5	29.165	0.446	27.512	−0.944	1.390	0.382	−1.390
CDK5R1	26.644	2.968	26.342	0.226	2.742	0.150	−2.742
SLC18A3	32.214	−2.603	30.672	−4.103	1.500	0.353	−1.500
CSNK1A1	27.723	1.889	26.998	−0.430	2.319	0.200	−2.319
CTSB	25.676	3.935	23.868	2.700	1.235	0.425	−1.235
CTSC	25.901	3.711	24.015	2.553	1.158	0.448	−1.158
CTSD	28.517	1.094	25.902	0.666	0.429	0.743	−0.429
CTSG	UDT		UDT				
BPTF	27.691	1.921	26.540	0.029	1.892	0.269	−1.892
GJB1	33.510	−3.899	27.844	−1.276	−2.623	6.160	2.623
GLS	28.279	1.333	27.666	−1.098	2.431	0.185	−2.431
GRIN1	25.319	4.292	25.904	0.664	3.629	0.081	−3.629
GRIN2A	28.312	1.299	28.594	−2.026	3.326	0.100	−3.326
GRIN2B	26.804	2.807	27.666	−1.098	3.905	0.067	−3.905
GRIN2D	27.508	2.103	27.559	−0.990	3.094	0.117	−3.094
GSK3B	24.337	5.274	24.018	2.550	2.724	0.151	−2.724
HSD17B10	26.251	3.361	26.386	0.182	3.178	0.110	−3.178
IDE	28.205	1.406	29.357	−2.788	4.194	0.055	−4.194
IFNG	UDT		UDT				

IL1A	33.345	−3.734	30.507	−3.939	0.205	0.867	−0.205
IL1B	33.710	−4.099	30.430	−3.862	−0.237	1.178	0.237
IL6	34.696	−5.085	31.522	−4.954	−0.131	1.095	0.131
INS	37.098	−7.487	33.078	−6.510	−0.977	1.968	0.977
INSR	29.899	−0.288	29.337	−2.769	2.481	0.179	−2.481
LRP1	25.850	3.762	25.255	1.313	2.449	0.183	−2.449
LRP2	31.533	−1.922	29.813	−3.245	1.324	0.400	−1.324
LRPAP1	26.140	3.471	26.979	−0.410	3.882	0.068	−3.882
MAPK1	26.430	3.181	26.554	0.014	3.167	0.111	−3.167
MAPK3	25.958	3.653	25.876	0.692	2.961	0.128	−2.961
MAPT	21.908	7.703	24.007	2.561	5.143	0.028	−5.143
MME	30.290	−0.678	28.025	−1.457	0.778	0.583	−0.778
NCSTN	26.632	2.979	26.759	−0.191	3.170	0.111	−3.170
PDE8B	31.316	−1.704	31.963	−5.395	3.691	0.077	−3.691
PSENEN	30.940	−1.329	30.820	−4.252	2.923	0.132	−2.923
PLD1	30.844	−1.232	29.824	−3.256	2.023	0.246	−2.023
PPP2CA	26.482	3.129	27.376	−0.808	3.938	0.065	−3.938
PRKACB	27.797	1.815	28.668	−2.100	3.915	0.066	−3.915
PRKCA	27.415	2.196	29.308	−2.740	4.936	0.033	−4.936
PRKCB	25.950	3.661	28.985	−2.417	6.078	0.015	−6.078
PRKCE	29.305	0.307	30.921	−4.352	4.659	0.040	−4.659
PRKCG	28.793	0.819	31.908	−5.340	6.159	0.014	−6.159
PKN1	24.437	5.174	26.669	−0.101	5.275	0.026	−5.275
PSEN1	27.528	2.084	28.668	−2.100	4.183	0.055	−4.183
PSEN2	30.881	−1.269	31.133	−4.565	3.296	0.102	−3.296
SERPINA3	24.698	4.913	23.956	2.612	2.301	0.203	−2.301
SNCA	24.339	5.273	26.813	−0.245	5.518	0.022	−5.518
SOAT1	29.900	−0.289	30.352	−3.784	3.495	0.089	−3.495
SOD2	24.152	5.460	24.590	1.978	3.481	0.090	−3.481
CAPNS2	30.751	−1.140	31.733	−5.165	4.025	0.061	−4.025
TNF	34.407	−4.796	34.430	−7.862	3.066	0.119	−3.066
UCHL1	23.404	6.207	26.828	−0.259	6.466	0.011	−6.466
VSNL1	28.551	1.060	33.327	−6.759	7.818	0.004	−7.818
GAL	29.532	0.079	30.895	−4.326	4.406	0.047	−4.406
ACHE	25.986	3.626	29.944	−3.375	7.001	0.008	−7.001
AGER	33.569	−3.957	33.062	−6.494	2.537	0.172	−2.537
NAE1	29.471	0.141	30.334	−3.765	3.906	0.067	−3.906
BCHE	33.601	−3.989	35.674	−9.106	5.117	0.029	−5.117
CAPNS1	24.751	4.861	25.683	0.886	3.975	0.064	−3.975
CHRNA7	29.695	−0.084	30.743	−4.174	4.091	0.059	−4.091

CSNK1D	23.968	5.644	26.147	0.421	5.222	0.027	−5.222
CYP46A1	34.434	−4.823	41.000	−14.432	9.609	0.001	−9.609
GAP43	24.446	5.165	28.034	−1.466	6.631	0.010	−6.631
GRIN2C	31.206	−1.595	41.000	−14.432	12.837	0.000	−12.837
SLC30A3	38.335	−8.723	41.000	−14.432	5.708	0.019	−5.708
ST6GAL1	29.525	0.086	32.496	−5.928	6.014	0.015	−6.014
UBQLN1	24.513	5.098	28.783	−2.215	7.313	0.006	−7.313

Table S3. Gene expression data and analysis for non−PD organoids and PD organoids infected with CHIKV obtained 14 days post inoculation. UDT: undetermined

	**non−PD**	**DCt**	**PD CHIKV**	**DCt**	**DDCt**	**fold change**	**log fold change**
Gene Symbol	CT		CT				
18S	4.024		16.565				
GUSB	22.349		27.891				
HPRT1	21.835		28.859				
GAPDH	16.749		23.526				
ABAT	22.019	0.330	27.652	0.239	0.091	0.939	−0.091
ACHE	25.873	−3.523	32.699	−4.808	1.285	0.410	−1.285
BSN	27.771	−5.421	33.529	−5.639	0.217	0.860	−0.217
CHAT	31.140	−8.790	32.772	−4.881	−3.909	15.021	3.909
CHRM1	30.327	−7.978	35.210	−7.319	−0.659	1.579	0.659
CHRM2	26.637	−4.287	30.496	−2.605	−1.682	3.209	1.682
CHRM3	24.891	−2.541	33.300	−5.409	2.868	0.137	−2.868
CHRM4	25.924	−3.574	32.125	−4.234	0.660	0.633	−0.660
CHRM5	28.195	−5.845	33.617	−5.727	−0.119	1.086	0.119
CHRNA2	31.187	−8.838	33.569	−5.679	−3.159	8.933	3.159
CHRNA3	27.334	−4.985	31.535	−3.644	−1.341	2.532	1.341
CHRNA4	28.765	−6.416	30.347	−2.457	−3.959	15.553	3.959
CHRNA5	27.223	−4.874	32.700	−4.809	−0.065	1.046	0.065
CHRNA6	28.147	−5.797	33.552	−5.662	−0.136	1.099	0.136
CHRNB2	27.406	−5.056	30.712	−2.821	−2.235	4.709	2.235
CHRNB3	28.456	−6.107	34.036	−6.145	0.038	0.974	−0.038
CHRNB4	25.612	−3.263	29.375	−1.485	−1.778	3.430	1.778
CHRND	30.959	−8.610	31.779	−3.889	−4.721	26.376	4.721
CHRNE	28.223	−5.874	36.525	−8.635	2.760	0.148	−2.760
COMT	21.685	0.664	32.470	−4.579	5.243	0.026	−5.243
CPLX1	27.547	−5.198	32.332	−4.441	−0.757	1.690	0.757

DRD2	25.649	−3.300	31.010	−3.119	−0.181	1.133	0.181
DRD3	36.286	−13.937	41.000	−13.109	−0.828	1.775	0.828
GABBR1	22.526	−0.177	25.862	2.029	−2.206	4.613	2.206
GABRA1	29.916	−7.566	34.206	−6.315	−1.251	2.380	1.251
GABRA2	33.256	−10.907	35.238	−7.348	−3.560	11.790	3.560
GABRA4	33.197	−10.847	35.484	−7.593	−3.254	9.542	3.254
GABRA5	28.907	−6.558	30.221	−2.330	−4.228	18.742	4.228
GABRA6	36.795	−14.446	41.000	−13.109	−1.337	2.526	1.337
GABRB1	27.857	−5.507	32.454	−4.563	−0.944	1.924	0.944
GABRB2	26.008	−3.659	34.744	−6.853	3.194	0.109	−3.194
GABRB3	24.540	−2.190	30.497	−2.606	0.416	0.750	−0.416
GABRD	29.867	−7.518	35.923	−8.033	0.514	0.700	−0.514
GABRE	26.860	−4.511	30.511	−2.620	−1.891	3.709	1.891
GABRG1	31.466	−9.117	34.730	−6.840	−2.277	4.848	2.277
GABRG2	27.734	−5.385	30.851	−2.960	−2.425	5.371	2.425
GABRG3	29.898	−7.548	33.147	−5.256	−2.292	4.899	2.292
GABRP	19.753	2.596	34.391	−6.501	9.097	0.002	−9.097
GABRQ	26.148	−3.799	29.819	−1.928	−1.870	3.656	1.870
GABRR1	31.249	−8.899	36.634	−8.743	−0.156	1.114	0.156
GABRR2	28.487	−6.138	34.463	−6.572	0.434	0.740	−0.434
GAD1	26.516	−4.167	31.970	−4.080	−0.088	1.063	0.088
GCH1	27.301	−4.952	32.409	−4.518	−0.434	1.351	0.434
GLRA1	29.351	−7.001	30.983	−3.093	−3.909	15.020	3.909
GLRA2	29.577	−7.228	27.889	0.002	−7.230	150.076	7.230
GLRA3	31.454	−9.104	31.165	−3.274	−5.830	56.885	5.830
GLRB	25.426	−3.077	29.358	−1.467	−1.610	3.053	1.610
GRIA1	27.155	−4.806	27.188	0.703	−5.509	45.527	5.509
GRIK4	30.909	−8.560	33.486	−5.595	−2.965	7.807	2.965
HTR1B	25.922	−3.572	31.236	−3.345	−0.227	1.171	0.227
HTR1E	25.996	−3.646	31.614	−3.723	0.077	0.948	−0.077
HTR2A	27.635	−5.286	29.740	−1.849	−3.437	10.827	3.437
HTR3A	32.637	−10.288	31.314	−3.423	−6.865	116.528	6.865
HTR3B	39.939	−17.590	41.000	−13.109	−4.481	22.329	4.481
HTR7	28.161	−5.811	36.188	−8.298	2.486	0.178	−2.486
MAOA	20.656	1.694	28.404	−0.513	2.207	0.217	−2.207
PHOX2A	29.938	−7.588	31.973	−4.082	−3.506	11.361	3.506
RIMS1	29.683	−7.334	34.553	−6.663	−0.671	1.592	0.671
RIMS3	27.063	−4.713	31.719	−3.829	−0.885	1.847	0.885
RIMS4	24.171	−1.822	27.578	0.313	−2.135	4.391	2.135
SLC18A2	27.918	−5.569	32.329	−4.438	−1.131	2.190	1.131
SLC18A3	28.925	−6.576	30.856	−2.965	−3.610	12.213	3.610

SLC1A3	22.752	−0.402	24.873	3.018	−3.420	10.702	3.420
SLC32A1	32.869	−10.520	41.000	−13.109	2.589	0.166	−2.589
SLC5A7	26.900	−4.550	28.373	−0.482	−4.068	16.777	4.068
SLC6A1	29.817	−7.468	32.843	−4.953	−2.515	5.716	2.515
SLC6A11	25.801	−3.452	33.219	−5.328	1.877	0.272	−1.877
SLC6A12	27.546	−5.197	34.648	−6.757	1.560	0.339	−1.560
SLC6A13	31.415	−9.066	37.453	−9.562	0.496	0.709	−0.496
SLC6A15	25.256	−2.907	30.466	−2.575	−0.332	1.259	0.332
SLC6A16	37.044	−14.695	36.867	−8.976	−5.719	52.666	5.719
SLC6A18	35.747	−13.398	41.000	−13.109	−0.289	1.222	0.289
SLC6A2	29.022	−6.672	37.711	−9.821	3.148	0.113	−3.148
SLC6A20	28.239	−5.890	33.407	−5.516	−0.374	1.296	0.374
SLC6A4	24.688	−2.339	36.171	−8.280	5.941	0.016	−5.941
SLC6A5	29.312	−6.963	35.186	−7.295	0.333	0.794	−0.333
SLC6A6	24.076	−1.726	29.763	−1.872	0.146	0.904	−0.146
SLC6A7	33.942	−11.593	41.000	−13.109	1.516	0.350	−1.516
SLC6A8	21.614	0.736	27.658	0.233	0.503	0.706	−0.503
SLC6A9	28.075	−5.726	41.000	−13.109	7.384	0.006	−7.384
SNPH	24.745	−2.396	41.000	−13.109	10.714	0.001	−10.714
STX1A	25.476	−3.126	33.485	−5.594	2.467	0.181	−2.467
STX1B	24.932	−2.582	34.159	−6.268	3.685	0.078	−3.685
STX3	23.130	−0.781	32.384	−4.493	3.712	0.076	−3.712
STXBP1	22.473	−0.124	30.438	−2.547	2.423	0.186	−2.423
SV2A	22.870	−0.520	31.799	−3.909	3.388	0.096	−3.388
SYN1	25.416	−3.067	32.353	−4.462	1.395	0.380	−1.395
SYN2	24.842	−2.493	32.353	−4.462	1.970	0.255	−1.970
SYN3	25.811	−3.462	32.501	−4.611	1.149	0.451	−1.149
SYP	23.301	−0.951	30.963	−3.072	2.121	0.230	−2.121
SYT1	24.036	−1.687	32.535	−4.644	2.958	0.129	−2.958
TPH1	21.969	0.380	31.888	−3.997	4.377	0.048	−4.377
18S	4.418		16.386				
GUSB	23.498		29.317				
HPRT1	23.007		30.427				
GAPDH	18.410		24.668				
IL1A	26.294	−2.795	31.867	−2.549	−0.246	1.186	0.246
IL1B	25.902	−2.404	31.447	−2.129	−0.275	1.210	0.275
IL2	37.521	−14.022	41.000	−11.683	−2.339	5.061	2.339
IL3	UDT		UDT				
IL4	UDT		UDT				
IL5	36.911	−13.413	36.810	−7.493	−5.920	60.534	5.920
IL6	28.923	−5.425	31.676	−2.359	−3.066	8.375	3.066

IL7	29.994	−6.496	32.541	−3.223	−3.273	9.665	3.273
IL8	23.324	0.174	28.371	0.947	−0.773	1.708	0.773
IL9	36.586	−13.088	41.000	−11.683	−1.405	2.648	1.405
IL10	29.127	−5.629	33.155	−3.838	−1.791	3.460	1.791
IL12A	31.476	−7.977	34.378	−5.061	−2.917	7.551	2.917
IL12B	36.211	−12.713	41.000	−11.683	−1.030	2.042	1.030
IL13	37.285	−13.786	41.000	−11.683	−2.104	4.298	2.104
IL15	31.824	−8.326	36.154	−6.836	−1.490	2.808	1.490
IL17A	UDT		UDT				
IL18	30.782	−7.284	36.352	−7.035	−0.249	1.189	0.249
CCL3	31.202	−7.704	24.971	4.346	−12.050	4239.804	12.050
CCL19	30.937	−7.438	28.579	0.739	−8.177	289.368	8.177
CCL2	22.749	0.750	25.861	3.456	−2.706	6.527	2.706
CCL5	29.127	−5.629	29.416	−0.099	−5.530	46.203	5.530
CCR2	38.102	−14.604	41.000	−11.683	−2.921	7.575	2.921
CCR4	30.485	−6.987	32.743	−3.425	−3.561	11.804	3.561
CCR5	34.342	−10.844	34.000	−4.682	−6.161	71.563	6.161
CCR7	29.918	−6.420	35.893	−6.576	0.156	0.897	−0.156
CXCR3	34.716	−11.217	41.000	−11.683	0.465	0.724	−0.465
CXCL10	27.561	−4.063	25.886	3.432	−7.495	180.338	7.495
CXCL11	28.559	−5.060	27.641	1.677	−6.737	106.647	6.737
CSF1	23.859	−0.360	27.029	2.289	−2.649	6.273	2.649
CSF2	30.007	−6.508	35.262	−5.945	−0.564	1.478	0.564
CSF3	28.321	−4.823	34.405	−5.088	0.265	0.832	−0.265
STAT3	21.286	2.213	24.523	4.794	−2.581	5.985	2.581
NFKB2	24.020	−0.521	28.233	1.084	−1.606	3.044	1.606
IKBKB	24.590	−1.092	33.437	−4.120	3.028	0.123	−3.028
CD3E	32.383	−8.884	41.000	−11.683	2.798	0.144	−2.798
CD4	28.869	−5.370	30.220	−0.902	−4.468	22.132	4.468
CD8A	28.828	−5.330	29.450	−0.133	−5.197	36.678	5.197
CD19	36.392	−12.893	41.000	−11.683	−1.210	2.314	1.210
IL2RA	34.444	−10.946	30.578	−1.261	−9.685	823.051	9.685
CD28	34.856	−11.358	30.558	−1.241	−10.117	1110.698	10.117
CD38	32.191	−8.693	31.756	−2.439	−6.254	76.339	6.254
CD40	26.560	−3.062	28.813	0.504	−3.566	11.843	3.566
PTPRC	32.506	−9.008	29.612	−0.294	−8.713	419.686	8.713
CD68	23.904	−0.405	23.530	5.788	−6.193	73.170	6.193
CD80	33.718	−10.220	34.401	−5.084	−5.136	35.154	5.136
CD86	34.604	−11.106	32.742	−3.424	−7.681	205.243	7.681
CTLA4	34.564	−11.065	39.481	−10.164	−0.902	1.868	0.902
CD40LG	39.231	−15.732	41.000	−11.683	−4.050	16.559	4.050

HLA−DRA	30.344	−6.846	29.706	−0.389	−6.457	87.851	6.457
HLA−DRB1	UDT		UDT				
TBX21	31.993	−8.495	35.355	−6.038	−2.457	5.491	2.457
TNFRSF18	30.559	−7.060	35.437	−6.119	−0.941	1.920	0.941
ICOS	36.160	−12.662	38.918	−9.601	−3.061	8.346	3.061
NOS2	31.609	−8.111	33.290	−3.973	−4.138	17.605	4.138
BCL2	24.410	−0.912	28.591	0.726	−1.638	3.112	1.638
BCL2L1	26.495	−2.997	30.875	−1.558	−1.439	2.711	1.439
BAX	21.558	1.940	25.836	3.481	−1.540	2.909	1.540
ICAM1	24.207	−0.708	28.615	0.703	−1.411	2.659	1.411
SELP	29.187	−5.689	35.572	−6.255	0.566	0.675	−0.566
SELE	28.415	−4.916	34.787	−5.470	0.554	0.681	−0.554
HMOX1	23.861	−0.363	28.658	0.660	−1.022	2.031	1.022
PTGS2	24.462	−0.964	30.188	−0.871	−0.093	1.066	0.093
LRP2	30.028	−6.529	30.431	−1.114	−5.416	42.685	5.416
CYP1A2	UDT		UDT				
CYP7A1	33.329	−9.830	41.000	−11.683	1.852	0.277	−1.852
IFNG	UDT		UDT				
PRF1	34.518	−11.020	37.594	−8.277	−2.743	6.695	2.743
GZMB	36.465	−12.967	33.517	−4.200	−8.767	435.623	8.767
GNLY	UDT		UDT				
FAS	24.398	−0.899	30.304	−0.987	0.088	0.941	−0.088
FASLG	UDT		UDT				
TGFB1	22.243	1.256	24.894	4.423	−3.168	8.986	3.168
SMAD3	23.706	−0.207	28.396	0.921	−1.128	2.186	1.128
SMAD7	24.199	−0.701	28.559	0.758	−1.459	2.748	1.459
SKI	21.226	2.272	25.644	3.673	−1.401	2.641	1.401
FN1	16.018	7.481	22.398	6.919	0.561	0.678	−0.561
C3	21.407	2.091	25.715	3.602	−1.511	2.850	1.511
TNF	29.850	−6.352	32.780	−3.463	−2.889	7.409	2.889
LTA	33.964	−10.465	35.222	−5.904	−4.561	23.601	4.561
ACE	28.256	−4.757	32.199	−2.882	−1.876	3.670	1.876
VEGFA	23.275	0.224	28.286	1.031	−0.807	1.750	0.807
CD34	28.925	−5.426	31.857	−2.539	−2.887	7.397	2.887
AGTR1	32.417	−8.918	41.000	−11.683	2.764	0.147	−2.764
AGTR2	30.021	−6.522	30.848	−1.531	−4.991	31.806	4.991
EDN1	23.585	−0.087	29.578	−0.261	0.174	0.886	−0.174
LIF	23.354	0.145	28.242	1.075	−0.931	1.906	0.931
LY96	28.819	−5.320	32.708	−3.391	−1.929	3.808	1.929
MIF	18.354	5.144	21.299	8.019	−2.874	7.332	2.874
NFATC3	23.310	0.188	26.465	2.852	−2.664	6.338	2.664

NFATC4	23.702	−0.204	28.665	0.652	−0.856	1.810	0.856
PF4	32.745	−9.246	35.909	−6.591	−2.655	6.297	2.655
SYK	25.367	−1.869	30.376	−1.059	−0.810	1.754	0.810
18S	4.975		13.717				
GUSB	24.178		26.568				
HPRT1	23.756		27.704				
GAPDH	18.910		22.247				
ABCA1	22.968	1.211	27.724	−1.156	2.367	0.194	−2.367
ADAM10	21.874	2.304	25.861	0.707	1.597	0.331	−1.597
ADAM17	24.215	−0.037	27.438	−0.870	0.832	0.562	−0.832
ADAM9	20.640	3.538	23.335	3.233	0.305	0.810	−0.305
APBA1	26.612	−2.434	27.853	−1.285	−1.149	2.218	1.149
APBA2	27.247	−3.069	28.970	−2.401	−0.667	1.588	0.667
APBA3	26.384	−2.205	29.356	−2.787	0.582	0.668	−0.582
APBB1	24.973	−0.795	26.765	−0.196	−0.599	1.514	0.599
APBB2	23.635	0.543	27.200	−0.632	1.175	0.443	−1.175
APBB3	26.463	−2.284	30.856	−4.288	2.004	0.249	−2.004
APCS	UDT		UDT				
APH1A	25.096	−0.918	25.708	0.860	−1.778	3.430	1.778
APH1B	24.753	−0.575	26.397	0.171	−0.746	1.677	0.746
APLP1	26.146	−1.968	28.392	−1.824	−0.144	1.105	0.144
APLP2	21.738	2.440	25.127	1.442	0.999	0.500	−0.999
APOE	20.729	3.449	24.510	2.059	1.390	0.381	−1.390
APP	18.600	5.578	20.806	5.762	−0.184	1.136	0.184
BACE1	24.263	−0.085	26.949	−0.381	0.296	0.815	−0.296
BACE2	22.657	1.521	24.270	2.299	−0.778	1.714	0.778
CAPN1	21.127	3.051	26.721	−0.153	3.204	0.109	−3.204
CASP3	25.482	−1.304	28.933	−2.365	1.061	0.479	−1.061
CASP6	24.998	−0.819	27.531	−0.963	0.143	0.905	−0.143
CDC2	25.776	−1.598	28.746	−2.178	0.580	0.669	−0.580
CDK5	25.157	−0.978	27.512	−0.944	−0.035	1.024	0.035
CDK5R1	25.552	−1.374	26.342	0.226	−1.600	3.031	1.600
SLC18A3	30.740	−6.562	30.672	−4.103	−2.459	5.498	2.459
CHRM1	32.370	−8.192	33.041	−6.473	−1.719	3.292	1.719
CHRM3	26.739	−2.560	30.713	−4.145	1.584	0.333	−1.584
CHRNA4	30.405	−6.226	28.907	−2.339	−3.888	14.801	3.888
CSNK1A1	24.199	−0.021	26.998	−0.430	0.409	0.753	−0.409
CTSB	20.521	3.658	23.868	2.700	0.957	0.515	−0.957
CTSC	19.266	4.912	24.015	2.553	2.359	0.195	−2.359
CTSD	20.005	4.173	25.902	0.666	3.507	0.088	−3.507
CTSG	UDT		UDT				

BPTF	23.545	0.633	26.540	0.029	0.605	0.658	−0.605
GJB1	29.543	−5.364	27.844	−1.276	−4.088	17.012	4.088
GLS	23.467	0.711	27.666	−1.098	1.809	0.285	−1.809
GRIN1	27.845	−3.666	25.904	0.664	−4.330	20.112	4.330
GRIN2A	28.442	−4.263	28.594	−2.026	−2.237	4.715	2.237
GRIN2B	30.698	−6.520	27.666	−1.098	−5.422	42.873	5.422
GRIN2D	24.497	−0.319	27.559	−0.990	0.672	0.628	−0.672
GSK3B	23.268	0.910	24.018	2.550	−1.640	3.118	1.640
HSD17B10	23.409	0.769	26.386	0.182	0.587	0.666	−0.587
IDE	25.395	−1.216	29.357	−2.788	1.572	0.336	−1.572
IFNG	UDT		UDT				
IL1A	26.896	−2.718	30.507	−3.939	1.221	0.429	−1.221
IL1B	26.574	−2.396	30.430	−3.862	1.466	0.362	−1.466
IL6	29.742	−5.563	31.522	−4.954	−0.610	1.526	0.610
INS	36.081	−11.902	33.078	−6.510	−5.392	42.000	5.392
INSR	24.664	−0.486	29.337	−2.769	2.283	0.206	−2.283
LRP1	20.966	3.212	25.255	1.313	1.899	0.268	−1.899
LRP2	30.842	−6.664	29.813	−3.245	−3.419	10.695	3.419
LRPAP1	22.717	1.461	26.979	−0.410	1.872	0.273	−1.872
MAPK1	24.405	−0.227	26.554	0.014	−0.241	1.182	0.241
MAPK3	23.677	0.501	25.876	0.692	−0.190	1.141	0.190
MAPT	24.924	−0.746	24.007	2.561	−3.307	9.896	3.307
MME	23.724	0.455	28.025	−1.457	1.912	0.266	−1.912
NCSTN	23.617	0.561	26.759	−0.191	0.752	0.594	−0.752
PDE8B	25.386	−1.208	31.963	−5.395	4.187	0.055	−4.187
PSENEN	31.480	−7.302	30.820	−4.252	−3.050	8.281	3.050
PLD1	24.803	−0.624	29.824	−3.256	2.631	0.161	−2.631
PPP2CA	22.875	1.304	27.376	−0.808	2.112	0.231	−2.112
PRKACB	25.885	−1.707	28.668	−2.100	0.393	0.761	−0.393
PRKCA	27.197	−3.018	29.308	−2.740	−0.279	1.213	0.279
PRKCB	25.911	−1.733	28.985	−2.417	0.684	0.622	−0.684
PRKCE	27.441	−3.263	30.921	−4.352	1.090	0.470	−1.090
PRKCG	30.863	−6.685	31.908	−5.340	−1.345	2.540	1.345
PKN1	22.940	1.238	26.669	−0.101	1.339	0.395	−1.339
PSEN1	24.909	−0.731	28.668	−2.100	1.368	0.387	−1.368
PSEN2	26.583	−2.405	31.133	−4.565	2.160	0.224	−2.160
SERPINA3	26.133	−1.955	23.956	2.612	−4.567	23.709	4.567
SNCA	23.510	0.669	26.813	−0.245	0.914	0.531	−0.914
SOAT1	25.510	−1.332	30.352	−3.784	2.452	0.183	−2.452
SOD2	21.755	2.423	24.590	1.978	0.445	0.735	−0.445
CAPNS2	23.103	1.075	31.733	−5.165	6.240	0.013	−6.240

TNF	30.548	−6.370	34.430	−7.862	1.492	0.356	−1.492
UCHL1	21.707	2.471	26.828	−0.259	2.730	0.151	−2.730
VSNL1	27.649	−3.471	33.327	−6.759	3.288	0.102	−3.288
GAL	29.142	−4.964	30.895	−4.326	−0.637	1.555	0.637
ACHE	24.642	−0.464	29.944	−3.375	2.912	0.133	−2.912
AGER	27.959	−3.780	33.062	−6.494	2.714	0.152	−2.714
NAE1	26.691	−2.513	30.334	−3.765	1.252	0.420	−1.252
BCHE	28.987	−4.809	35.674	−9.106	4.297	0.051	−4.297
CAPNS1	20.366	3.812	25.683	0.886	2.927	0.132	−2.927
CHRNA7	28.672	−4.493	30.743	−4.174	−0.319	1.247	0.319
CSNK1D	21.767	2.411	26.147	0.421	1.990	0.252	−1.990
CYP46A1	35.514	−11.335	41.000	−14.432	3.096	0.117	−3.096
GAP43	24.747	−0.568	28.034	−1.466	0.898	0.537	−0.898
GRIN2C	27.815	−3.637	41.000	−14.432	10.795	0.001	−10.795
SLC30A3	33.262	−9.083	41.000	−14.432	5.348	0.025	−5.348
ST6GAL1	28.664	−4.486	32.496	−5.928	1.442	0.368	−1.442
UBQLN1	22.741	1.438	28.783	−2.215	3.653	0.080	−3.653
